# PGPB-driven bioenrichment and metabolic modulation of *Salicornia europaea* under marine Aquaponic conditions

**DOI:** 10.1007/s11274-025-04335-5

**Published:** 2025-04-07

**Authors:** Maria J. Ferreira, Erika Garcia-Cardesín, I. Natalia Sierra-Garcia, Diana C. G. A. Pinto, Javier Cremades, Helena Silva, Ângela Cunha

**Affiliations:** 1 Department of Biology & Center for Environmental and Marine Studies (CESAM), University of Aveiro, Campus de Santiago, Aveiro, 3810-193 Portugal; 2 LAQV-REQUIMTE & Department of Chemistry, University of Aveiro, Campus de Santiago, Aveiro, 3810-193 Portugal; 3 Interdisciplinary Center for Chemistry and Biology (CICA), University of A Coruña, A Coruña, 15071 Spain

**Keywords:** *Brevibacterium casei EB3*, IMTA, PGPB, *Pseudomonas oryzihabitans RL18*, Secondary metabolites, Metabolic pathways

## Abstract

This study analyzed the secondary metabolite profile of *Salicornia europaea* inoculated with *Brevibacterium casei* EB3 and *Pseudomonas oryzihabitans* RL18 in aquaponic systems, exploring the metabolic mechanisms responsible for the observed shifts. Experiments were conducted in both microcosm and pilot-scale aquaponic setups to evaluate how these metabolic shifts vary across different system scales and their potential contributions to the observed increased accumulation of bioactive compounds with antioxidant and antimicrobial properties, including some phenolic acids, such as caffeic acid (154-fold), flavonoids (2.85-fold), and some unsaturated fatty acids, such as oct-3-enoic acid (32-fold). Metabolic profiling revealed shifts in pathways associated with plant growth and stress resilience, such as amino acid and phenolic biosynthesis. Additionally, differences in metabolic responses observed between microcosm and pilot-scale systems underscored the importance of understanding scaling effects. These findings highlight the potential for optimizing aquaponic systems by leveraging microbial-plant interactions to enhance ecological and economic outcomes. This approach offers valuable applications in nutrient recycling, phytopharmaceutical development, and the advancement of saline agriculture within integrated aquaculture frameworks.

**Supplementary Information:**

The online version contains supplementary material available at 10.1007/s11274-025-04335-5.

## Introduction

Increasing environmental pressures, such as soil salinization and drought, significantly affect traditional food production methods, raising concerns about environmental degradation, food security, and socioeconomic stability (Halpern et al. 2022). These challenges create opportunities to explore novel strategies and advance alternative production systems, such as saline agriculture and integrated multi-trophic aquaculture (IMTA) (Tuomisto et al. 2017; Halpern et al. 2022).

Within this context of exploring alternative food production systems, *Salicornia europaea* (syn. *S. ramosissima*) has emerged as a promising candidate for marine IMTA systems. In these systems, *S. europaea* functions as a sustainable, high-value crop with significant market potential, thriving alongside aquaculture species (Sharma et al. 2016; Castilla-Gavilán et al. 2024; Ferreira et al. 2024). Its exceptional salt tolerance and ability to thrive in coastal environments make it particularly suitable for these systems. This plant acts as a biofilter that absorbs excess nutrients, improving water quality and boosting its nutritional profile (Webb et al. 2012; Jerónimo et al. 2021).

The success of *S. europaea* in these systems is strongly linked to its interactions with root-associated microorganisms, which are critical for plant health and nutrient uptake (Philippot et al. 2013). These microorganisms can improve plant nutrition, resistance to abiotic stress, and host defense (Hayat et al. 2010; Chiaranunt and White 2023). In traditional soil-based systems, the rhizosphere forms a dynamic environment where root exudates enrich microbial communities (Badri and Vivanco 2008; Haldar and Sengupta 2015; Lynch and Neufeld 2015). However, in soilless aquaponic systems, the dilution of root exudates and increased microbial mobility, as reported by Lobanov et al. (2022), pose unique challenges that can destabilize these beneficial interactions (Lobanov et al. 2022).

Plant growth-promoting bacteria (PGPB) play a pivotal role in plant-microbiome communication by inducing metabolic reprogramming in their hosts, influencing both primary and secondary metabolite pathways (Pang et al. 2021; Ferreira et al. 2023, 2024). PGPB can enhance the synthesis of precursors for defense-related secondary metabolites and modulate primary metabolic pathways, redirecting resources toward stress resistance mechanisms (Mhlongo et al. 2020). This metabolic reconfiguration offers substantial economic potential, enhancing the antioxidant, anti-inflammatory, and antimicrobial properties of plants, making them valuable for various applications (Bakaeva et al. 2024; Puccinelli et al. 2024).

Previous studies have demonstrated that PGPB can significantly enhance the growth and nutrient extraction of *S. europaea* under both soil-based and aquaponic conditions (Ferreira et al. 2024, 2025). However, while these investigations provided valuable insights into biomass accumulation and nutrient uptake, the impact of PGPB on the plant’s secondary metabolite profile in aquaponic systems remains largely unexplored—a critical gap given the potential of these metabolites to boost the nutritional and pharmaceutical value of *S. europaea*.

Building on prior aquaponic research, our study focuses exclusively on the enrichment and expression of secondary metabolites in *S. europaea* under aquaponic conditions—a context that has not been previously addressed. By integrating insights from marine microbiology and plant biotechnology, our innovative approach aims to elucidate how targeted PGPB applications modulate metabolic pathways and enhance the production of bioactive compounds. Importantly, this work distinguishes itself by concentrating on secondary metabolite production rather than on biomass or nutrient extraction, thereby offering a transformative strategy for sustainable saline agriculture and IMTA systems, with significant implications for environmental resilience, food security, and the development of natural bioactive compounds.

## Materials and methods

### Plant inoculation and growth

The inoculation and cultivation of *Salicornia europaea* followed protocols adapted from previously published methods (Ferreira et al. 2023), ensuring reproducibility while accommodating experimental specificities. Seeds from production fields at Horta dos Peixinhos (Aveiro, Portugal) were inoculated with the plant growth-promoting bacteria (PGPB) *Brevibacterium casei* EB3 and *Pseudomonas oryzihabitans* RL18. These isolates can produce siderophores, ACC deaminase, indole-3-acetic acid, exopolysaccharides, and cellulases. In addition, *B. casei* EB3 produces proteases and can fix atmospheric nitrogen, while *P. oryzihabitans* RL18 produces amylases and is able to solubilize phosphate (see Supplementary Table S1). These strains were previously isolated from the root endosphere and rhizosphere of *Salicornia europaea* plants collected from various locations along the Portuguese coast (Ferreira et al. 2021). Bacterial strains were cultured in Tryptic Soy Broth (TSB; Liofilchem, Roseto degli Abruzzi) supplemented with 25 g L⁻¹ NaCl (30 °C ± 2 °C for 48 h), harvested by centrifugation (5000 *g*, 10 min), washed three times in sterile saline solution (NaCl 9 g L^-1^) and finally resuspended in sterile saline solution to a density of 10⁸ CFU mL⁻¹. Equal volumes of each suspension were mixed for co-inoculation. Seeds were surface-sterilized (immersion, for 2 min., in 1 mL solution of 1:1 proportion of hydrogen peroxide (30%) and ethanol (96%), followed by rinsing three times with sterile distilled water). The absence of residual microbes was confirmed by plating a subsample of the sterilized seeds on TSA medium amended with 25 g L⁻¹ NaCl. These plates were incubated for 48 h at 30 °C ± 2 °C, and no microbial growth was observed, indicating that the sterilization protocol was effective (Ferreira et al. 2023).

Seeds were then immersed in the appropriate inoculum for 2 h with agitation (150 rpm, 30 °C± 2 °C), and subsequently air dried in a laminar flow chamber. Non-inoculated seeds (controls) were treated similarly using sterile saline solution. To verify that the bacteria successfully attached to the seed surface, a subsample of the inoculated seeds was plated on TSA supplemented with 25 g L⁻¹ NaCl. After 48 h of incubation at 30 °C ± 2 °C, the characteristic colonies of each isolate were recovered, confirming efficient microbial attachment (Ferreira et al. 2023).

Simultaneous aquaponics experiments were conducted at microcosm (300 mL pots) and pilot-scale (100 L tanks) levels. Seedlings germinated on 1% agar plates (Sanyo MLR 350 H Versatile Environmental Test Chamber, Moriguchi, Osaka, Japan) under controlled light (350 µmol m^-2^ s^-1^ photon flux density), temperature (24 °C), and photoperiod (16/8 h) conditions were transplanted to perlite-filled pots irrigated with gradually salinized Hoagland’s solution (gradual increase to 10 g L^-1^ by adding 2.5 g L^-1^ of commercial marine salt every four days, during 15 days). Plants were kept under these conditions for three months and were reinoculated with bacterial suspensions or sterile saline on days 45 and 85 post-transplant (5 mL of the appropriate bacterial solution /sterile saline solution for inoculated and control plants, respectively).

After three months, plants were acclimated to diluted seawater (first week, 15‰ with tap water added with 0.5 mL L^− 1^ of an oligo-elements solution - composition for 1 L of solution: Na_2_EDTA·2H_2_O, 14 g; Fe(NH_4_)_2_(SO_4_)_2_·6H_2_O, 14 g; MnSO_4_·4H_2_O, 1.6 g; FeCl_3_·6H_2_O, 0.5 g; ZnSO_4_·7H_2_O, 0.2 g; CoSO_4_·7H_2_O, 0.05 g; second and third weeks, salinity was increased to 20‰). Salinity was confirmed with a hydrotherm densimeter (Nahita 1000–1100 Kg m^− 3^). Both setups maintained continuous aeration and a consistent photoperiod (16/8 h), temperature (20 °C ± 2 °C), and light intensity (350 µmol m^− 2^ s^− 1^ photon flux density).

In the microcosm set-up, ten plants for each inoculation condition (NI - non-inoculated plants; EB3 – plants inoculated with *B. casei* EB3, RL18 – plants inoculated with *P. oryzihabitans* RL18, and EB3 + RL18 – plants inoculated with both bacteria) were individually placed in 300 mL aquaponics units inside in a growth chamber (Liebherr^®^) with shaking for medium homogenization (Supplementary Figure S1). In the pilot-scale experiment, due to a limited number of available tanks (6), we evaluated only the EB3 + RL18 formulation as the inoculum. In this trial, 24 inoculated and 24 non-inoculated control plants were distributed across six aquaponic units (three 100-L tanks per treatment, with eight plants per tank) under continuous aeration. Plants were placed on a polystyrene floater for mechanical support, ensuring that the whole root system was permanently immersed (Supplementary Figure S1). Plants were kept in these set-ups for two months, until the end of the experiment.

### Sample Preparation for phytochemical analyses

Aboveground biomass, dried at 60 °C and sampled in triplicate (per treatment) was ground into a fine powder under liquid nitrogen. Samples were prepared for GC-MS and UHPLC-MS analyses using specific protocols detailed below.

### Gas chromatography-mass spectrometry (GC-MS)

To enhance sensitivity, 10 mg of powdered sample of each biological replicate was derivatized in a solution containing 125 µL of pyridine, 125 µL of N, O-bis(trimethylsilyl)trifluoroacetamide (BSTFA), 25 µL of trimethylsilyl chloride (TMSCl), and 90 µL of hexatriacontane (1 mg mL⁻¹, internal standard), with dichloromethane added to 1 mL total volume. The mixture was incubated at 70 °C for 40 min with stirring, cooled, filtered through a 0.45 μm nylon filter, and injected into a Shimadzu QP2010 Ultra GC-MS equipped with a ZB-5 ms capillary column (30 m × 0.25 mm × 0.25 μm). The injector and transfer line temperatures were set at 320 °C and 200 °C, respectively. The column was programmed from 70 °C to 300 °C in stepwise increments, using helium as the carrier gas at 1.19 mL min⁻¹. The detailed description of this procedure can be found in Ferreira et al. (2024) (Ferreira et al. 2024). Metabolite identification was performed using NIST14 and Wiley libraries, and quantification relied on calibration curves created with authentic standards (Ferreira et al. 2024).

### Ultra-high-performance liquid chromatography-mass spectrometry (UHPLC-MS)

Ethanol extracts of each biological replicates were prepared by mixing 10 mg of sample powder with 2 mL of ethanol (two 24-hour cycles). Extracts were carefully transferred to glass tubes, and dried under vacuum at 40 °C, and resuspended in methanol (10 mg mL⁻¹). Filtrates (0.2 μm nylon) were analyzed on a Thermo Scientific Ultimate 3000 RSLC UHPLC system coupled to an LTQ XL mass spectrometer. Separation was achieved on a Hypersil GOLD column (100 mm × 2.1 mm, 1.9 μm) at 30 °C, using a mobile phase of 0.1% formic acid. A solvent gradient from 5 to 100% over 33 min was employed at a flow rate of 0.2 mL min⁻¹. Detection was conducted at 320 nm, and the mass spectrometer operated in negative-ion mode, covering m/z 50–2000. The detailed method can be found in Ferreira et al. (2024) (Ferreira et al. 2024). Quantification used calibration curves generated with authentic standards prepared in methanol (Ferreira et al. 2024).

### Statistical analyses

Normality was assessed by the Shapiro-Wilk test. Differences between experimental conditions were evaluated using a t-test or one-way ANOVA whenever normal distribution was verified. Non normal distributions were evaluated by the Mann-Whitney or Kruskal-Wallis test. Significant differences were always considered at *p* < 0.05.

All the above-mentioned statistical analyses were performed using IBM SPSS statistics, version 28.0.1.1 (14).

Log_2_ fold change of two groups’ means was calculated using Microsoft^®^ Excel for Mac (version 16.83).

## Results

The phytochemical characterization of aquaponic-grown plants was based on GC-MS and UHPLC-MS analysis, which detected a wide variety of metabolites. The quantification of all the detected metabolites, either individually or grouped by chemical classes is represented in Supplementary Tables S2-S6 and in Supplementary Figures S2 and S3.

**Fig. 1:**
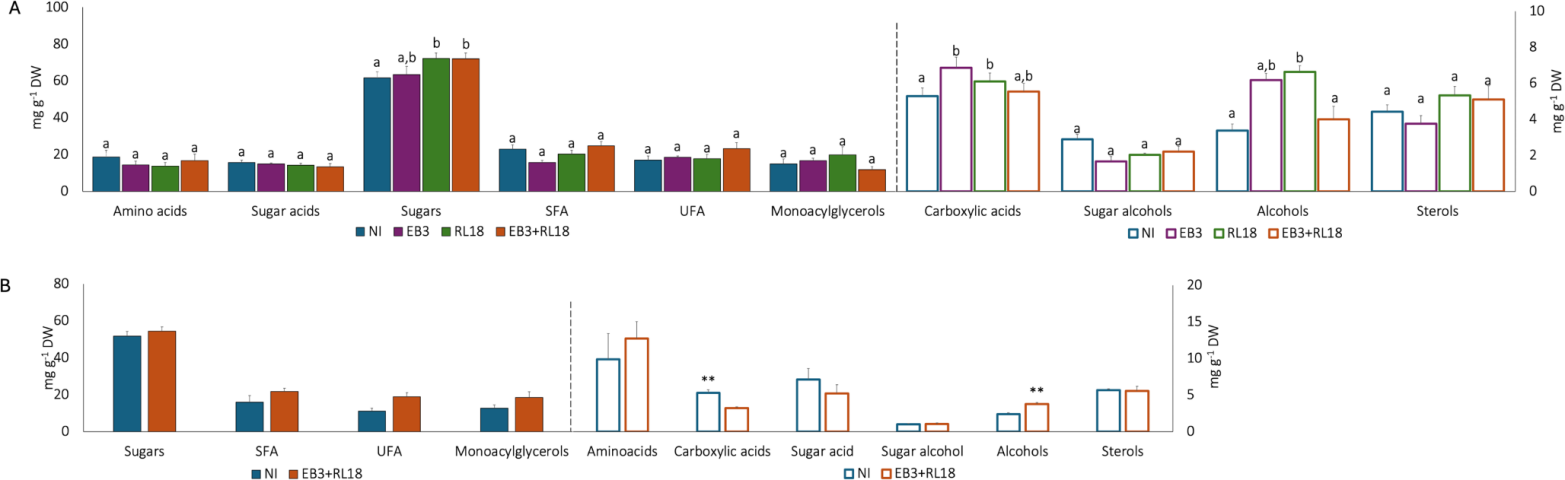
Phytochemical profile of plants grown in aquaponic microcosm (**A**) and pilot-scale (**B**) conditions, according to chemical families of compounds detected by GC-MS. NI- non-inoculated plants; EB3 – plants inoculated with *Brevibacterium casei* EB3; RL18 – plants inoculated with *Pseudomonas oryzihabitans* RL18; EB3 + RL18–plants inoculated with *Brevibacterium casei* EB3 and *Pseudomonas oryzihabitans* RL18. The columns represent the average of three biological replicates, and the error bars correspond to the standard error. Data were compared by Kruskal-Wallis test (microcosm conditions) and Mann-Whitney U test (pilot-scale conditions). Different letters (a-b, *p* < 0.05) or * (*p* < 0.05) and ** (*p* < 0.005) represent statistically significant differences in metabolite concentrations among plants

Considering broad compound classes, inoculation under aquaponic conditions resulted in a notable increase in carboxylic acids and alcohols. In pot-grown plants, single inoculation with *B. casei* EB3 led to carboxylic acid accumulation of 6.9 ± 0.6 mg g⁻¹ DW, while inoculation with *P. oryzihabitans* RL18 resulted in 6.1 ± 0.5 mg g⁻¹ DW. Alcohol content was similarly elevated in pot-grown plants inoculated with *P. oryzihabitans* RL18 (6.6 ± 0.3 mg g⁻¹ DW) and in tank-grown plants subjected to co-inoculation (3.8 ± 0.2 mg g⁻¹ DW), compared to non-inoculated controls (Supplementary Table S2 and Fig. 1A and B, respectively, for pots and tanks).

Interestingly, non-inoculated tank plants accumulated higher levels of carboxylic acids (5.3 ± 0.4 mg g⁻¹ DW) compared to inoculated tank plants (3.2 ± 0.2 mg g⁻¹ DW). Additionally, the total sugar content of pot-grown plants inoculated with strain RL18, either alone (72.1 ± 3.0 mg g⁻¹ DW) in pots or in combination with EB3 (72.0 ± 3.2 mg g⁻¹ DW), in tanks, was significantly higher than that of non-inoculated plants (Supplementary Table S2 and Fig. 1A and B, respectively, for pots and tanks).

In a more detailed analysis, considering each detected compound, more differences emerged. Some metabolites were exclusive to particular inoculation conditions. In pots (Supplementary Tables S2, S3 and S5), borneol, a monoterpenoid, was only detected in inoculated plants. Tocopherol, another compound of interest, was found only in plants inoculated with both bacteria. In comparison to non-inoculated plants, plants inoculated with *P. oryzihabitans* RL18 showed significantly higher content in interesting compounds such a psicose, 1-eicosanol, docosanol and gorgosterol than all other plants. Plants inoculated with *B. casei* EB3 alone, accumulated significantly more butanoic acid than control plants, and more monostearin than plants inoculated with the association EB3 + RL18. EB3 inoculated plants also accumulated more eicos-11-enoic acid than all the other groups. Inoculation with the mixture of the two PGPB, caused a significant increase in stearic acid and in ergosterol. Cholesterol was only detected in non-inoculated plants. Control plants also exhibited higher levels of succinic acid and xylose than all remaining groups.

In tanks (Supplementary Tables S2, S4 and S6), non-inoculated plants had higher levels acetic, lactic, caproic and glyceric acids and stigmastanol than inoculated plants. On the contrary, inoculated plants had higher levels of eicosanol, docosanol, stearic acid, oct-3-enoic acid and gorgosterol than non-inoculated controls. Like in the pot experiments, only inoculated plants produced detectable amounts of tocopherol and borneol. Ephedrine, an alkaloid, was also found exclusively in inoculated plants.

**Fig. 2:**
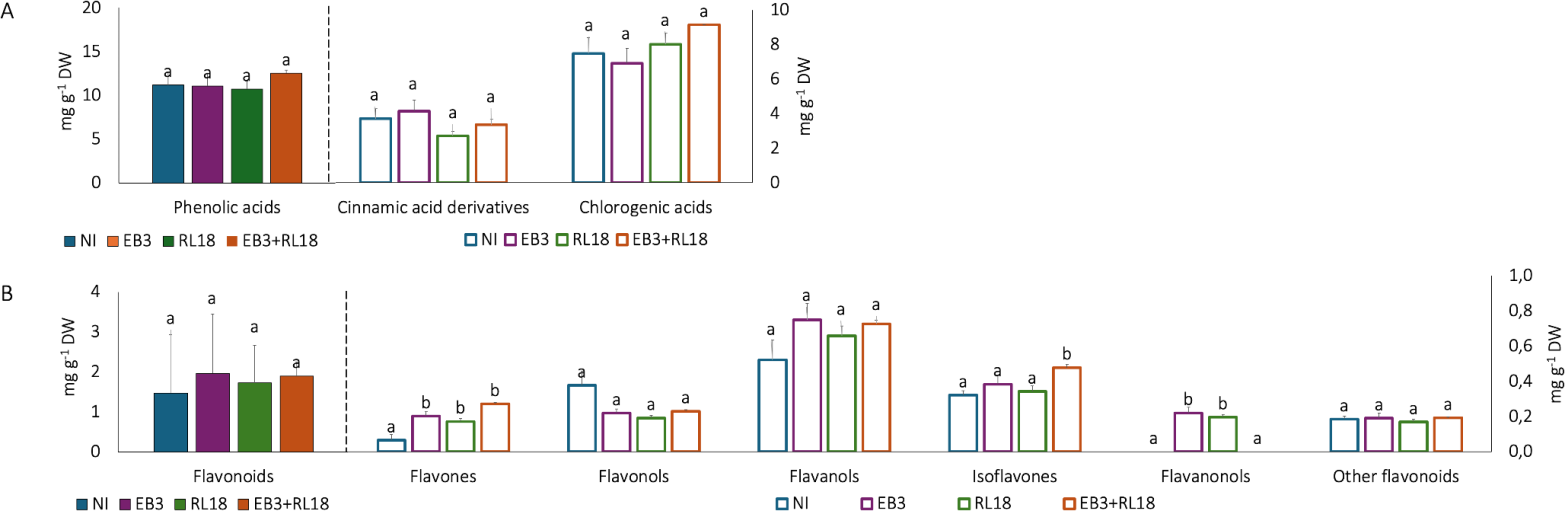
Phytochemical profile of plants grown in aquaponic microcosm conditions, according to chemical families of compounds detected by UHPLC-MS (**A**. Phenolic acids; **B** – Flavonoids). NI- non-inoculated plants; EB3 – plants inoculated with *Brevibacterium casei* EB3; RL18 – plants inoculated with *Pseudomonas oryzihabitans* RL18; EB3 + RL18–plants inoculated with *Brevibacterium casei* EB3 and *Pseudomonas oryzihabitans* RL18. The columns represent the average of three biological replicates, and the error bars correspond to the standard error. Data were compared by Kruskal-Wallis test. Different letters (a-b) represent statistically significant differences (*p* < 0.05) in metabolite concentrations among plants

Although the levels of total phenolic acids and flavonoids were similar between pot-grown plants regardless of their inoculation status, a more detailed analysis revealed interesting differences (Fig. 2A and B, Supplementary Tables S2, S5, and Supplementary Figure S2). Significant variations were observed in specific flavonoid subclasses: flavones showed increased concentrations under all inoculation conditions, isoflavones were elevated in plants co-inoculated with *B. casei* EB3 and *P. oryzihabitans* RL18, and flavanonols were significantly higher in plants inoculated with either EB3 or RL18 individually.

Two chlorogenic acids, feruloylquinic acid and caffeoyl-feruloylquinic acid, were only produced by inoculated plants (the former only by plants inoculated with *P. oryzihabitans* RL18). Ononin, a formononetin derivative, norbellidifodin (xanthone), sinapaldehyde and luteolin were only detected in inoculated plants. Taxifolin and was only detected in plants inoculated with *B. casei* EB3, or with EB3 + RL18. 5-hydroxyl-6,8-dimethoxy-7-hexoside flavone was only detected in plants inoculated with EB3 + RL18, which also accumulated formononetin (*p* = 0.04), compared to non-inoculated plants. On the other hand, isorhamnetin and piceid, a stilbenoid, were only detected in non-inoculated plants, which were also enriched in quercetin.

**Fig. 3:**
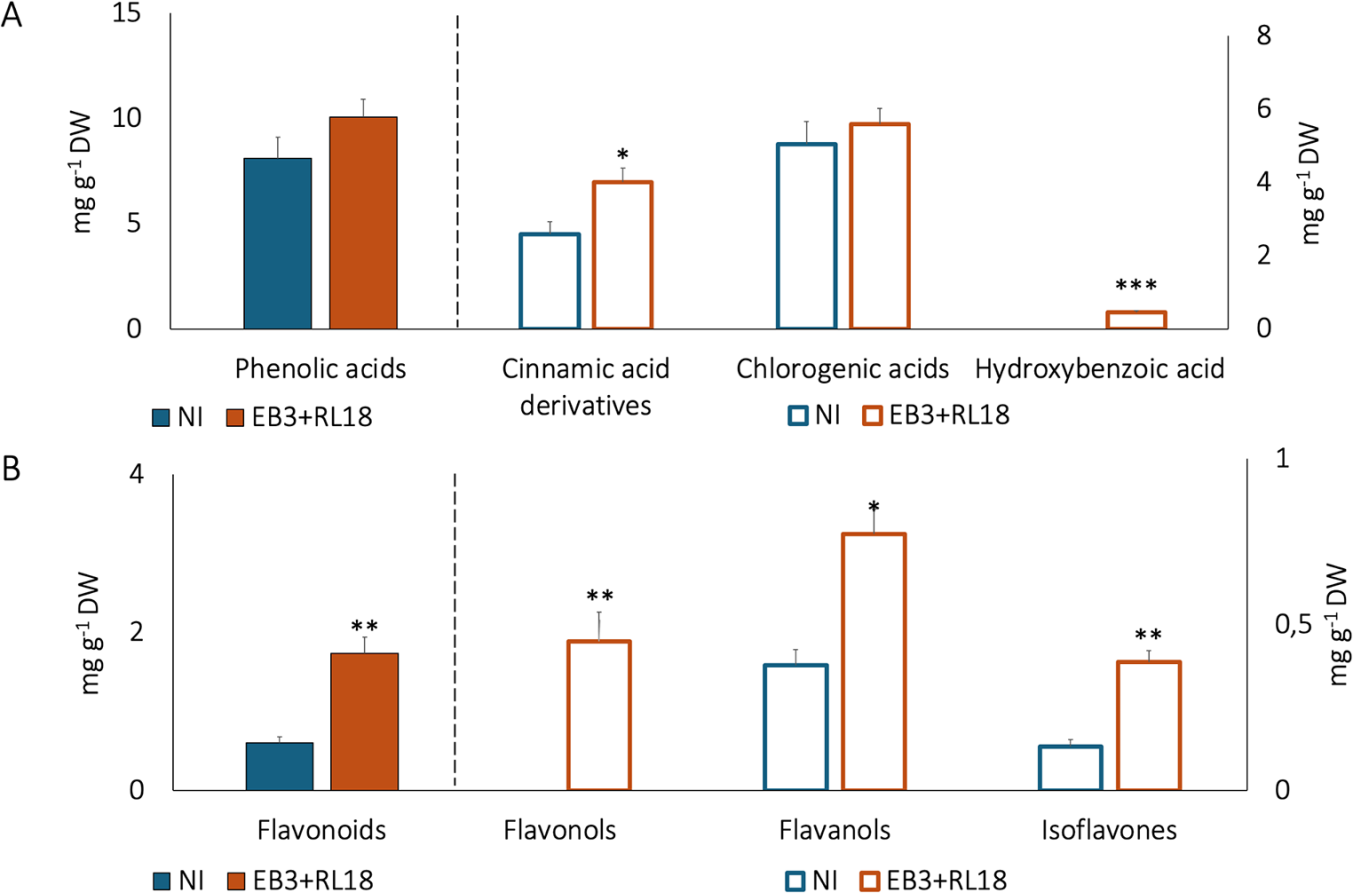
Phytochemical profile of plants grown in aquaponic pilot-scale conditions, according to chemical families of compounds detected by UHPLC-MS (**A**. phenolic acids; **B** – Flavonoids). NI- non-inoculated plants; EB3 + RL18–plants inoculated with *Brevibacterium casei* EB3 and *Pseudomonas oryzihabitans* RL18. The columns represent the average of three biological replicates, and the error bars correspond to the standard error. Data were compared by Kruskal-Wallis test Data were compared by Mann-Whitney U test. * (*p* < 0.05), ** (*p* < 0.005) and *** (*p* < 0.001) represent statistically significant differences in metabolite concentrations among plants

The phenolic content of inoculated tank-plants (Fig. 3A and B, Supplementary Tables S2, S6, and Supplementary Figure S3) exhibited a distinct pattern compared to the control plants. Inoculated plants accumulated 2.8 times more total flavonoids than the controls, with significant differences revealing enhanced accumulation across all flavonoid classes detected. Notably, specific flavonoids such as gallocatechin, quercetin, luteolin, kaempferol, and acacetin were exclusively produced by the inoculated plants, as well as formononetin.

In specific phenolic acids, some differences were also detected (Supplementary Figure S3). Inoculation caused an enrichment in *p*-coumaric acid and the exclusive production of caffeic acid, feruloylquinic acid, hydroxybenzoic acid and 1,3-*O*-feruloyl-caffeoylglycerol, a phenylpropane glyceride. Cinnamic acid was only detected in non-inoculated controls.

Borneol, tocopherol, medioresinol, norbellidifodin, piceid and sinapaldehyde were not quantified due to the lack of an appropriate standard.

**Fig. 4:**
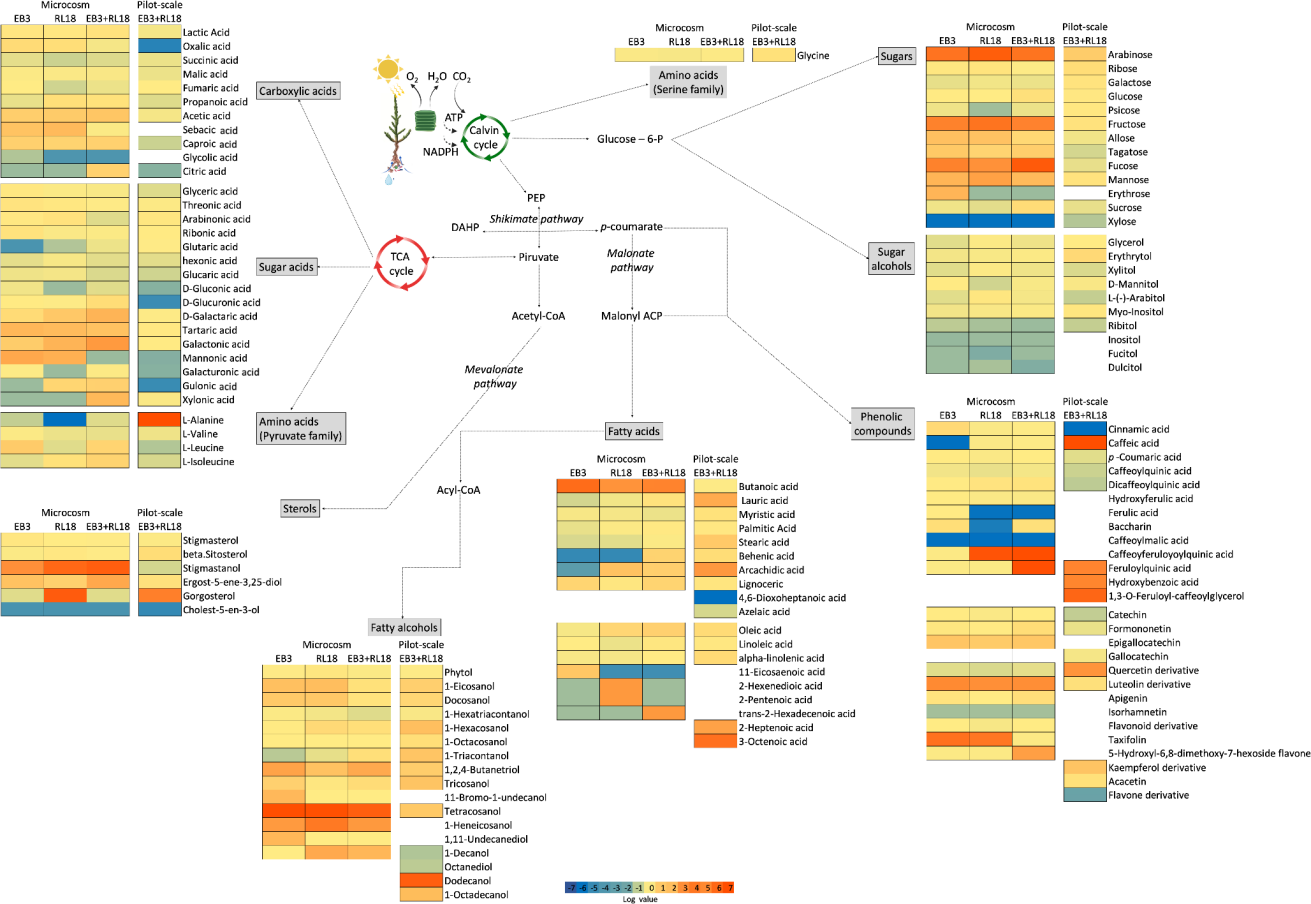
General overview of the shifts in the metabolite profile of *Salicornia europaea* grown in aquaponic systems (microcosm or pilot-scale), upon inoculation with PGPB, and their relation with metabolic pathways. For each metabolite, the level of variation [expressed as log_2_ (EB3/control or RL18/control or EB3 + RL18/ control)] is represented as a heatmap. EB3 – plants inoculated with *Brevibacterium casei* EB3; RL18 – plants inoculated with *Pseudomonas oryzihabitans* RL18, EB3 + RL18 – plants inoculated with *Brevibacterium casei* EB3 and *Pseudomonas oryzihabitans* RL18; PEP – phosphoenolpyruvate; DAHP − 3-deoxy-D- arabino -heptulosonic acid 7-phosphate

In order to infer on biochemical pathways that may have involved a metabolic shift, metabolites were organized according to main plant physiological process and biochemical patways, and up or downregulation was represented in the form of heatmaps (Fig. 4). For each metabolite, the level of variation was expressed as log_2_ (EB3/control or RL18/control or EB3 + RL18/ control).

In pot-grown plants inoculated with the two PGPB, there was an overexpression of sugars (e.g. arabinose, fucose, fructose), fatty acids (e.g. saturated butanoic acid, and unsaturated hex-2-enedioic, pent-2-enoic, *trans*-hexadec-2-enoic acids), sterols (e.g. stigmastanol, gorgosterol), alcohols (e.g. tetracosanol, decan-1-ol), and phenolic compounds [e.g. caffeic feruloylquinic acid (CFQA), feruloylquinic acid (FQA), luteolin, taxifolin]. On the other hand, inoculation of pot plants was associated with a decrease in glycolic and glutaric acids, alanine, cholesterol, xylose, caffeic acid and isorhamnetin. The majority of sugar alcohols were also related to a negative profile response to inoculation in microcosm conditions.

Inoculated plants grown in the tanks showed a profile response characterized by an increase in alanine, dodecanol, oct-3-enoic acid, gorgosterol, and phenolic compounds (e.g. caffeic acid, 1,3-*O*-feruloyl-caffeoylglycerol, feruloylquinic acid, quercetin, kaempferol) compared to non-inoculated plants. Some metabolites were, however, associated with a negative profile response, characterized by a decrease in the concentration of oxalic acid, glucuronic acid, sugar alcohols, and the phenolic compounds, cinnamic acid and a flavone derivative.

## Discussion

The current study demonstrates the profound impact of PGPB inoculation on the metabolic profile of *Salicornia europaea* cultivated under aquaponic conditions. Our findings reveal significant metabolic reprogramming that enhances the plant’s physiological adaptation, stress resilience, and accumulation of valuable bioactive compounds. These changes not only improve the plant’s growth and stress tolerance but also have implications for its potential nutraceutical and pharmacological applications.

### Enrichment of key compounds implies metabolic reprogramming for physiological adaptation

Our analysis revealed a significant plant metabolic shift caused by PGPB inoculation. It was characterized by an enrichment in essential compounds that fundamentally altered the physiological state of the plant and their adaptive responses.

The inoculation notably enhanced amino acid biosynthesis, particularly alanine, while several others like threonine, asparagine, proline, lysine, and tyramine were exclusively detected in inoculated plants. This suggests a broad enhancement of amino acid metabolism. Amino acids are crucial for plant growth, functioning as building blocks for proteins and enzymes that regulate intracellular processes (Lopez and Mohiuddin Shamin 2024). The enhanced biosynthesis likely reflects an increased demand for macronutrients, especially nitrogen and phosphorus, which are essential for amino acid production (Elshamly et al. 2024). Thus, it appears that the stimulation of amino acid synthesis leads to a higher need for these macronutrients, which in turn could support elevated metabolic activity and amino acid production. This creates a feedback loop where the need for macronutrients drives the plant to enhance nutrient uptake, further supporting the metabolic shift and amino acid biosynthesis. Such nutrient–metabolite feedback interactions have been well-documented in plant metabolism (Gupta et al. 2024).

Furthermore, amino acid upregulation may be linked to the biosynthesis of phenolic compounds and antioxidants like glutathione, boosting plant growth and antioxidant capacity (Hasanuzzaman et al. 2017; Saltveit 2018). Via the shikimic acid pathway, phosphoenolpyruvate (PEP) is involved in the synthesis of phenylalanine and tyrosine that contribute to the synthesis of various phenolic compounds, including phenolic acids and flavonoids (Falcone Ferreyra et al. 2012; Anantharaju et al. 2016; Saltveit 2018). Through pathways such as the shikimic acid and malonate pathways, plants increased levels of phenolic compounds that contribute to biomass production, as they correlate with higher fiber content (e.g. lignin) and other polymers (e.g. suberin and cutin) essential for sustaining plant growth and stress resistance (Saltveit 2018; Schefer et al. 2021). Flavonoid synthesis can also be upregulated *via* the mevalonic acid pathway(Zähringer et al. 1978). These metabolic shifts—characterized by increased production of amino acids and phenolic compounds—may be linked to siderophore production by *Pseudomonas oryzihabitans* RL18, which enhances iron uptake and supports overall plant metabolism (Ferreira et al. 2021). Siderophore synthesis demands substantial amounts of amino acids such as lysine, threonine, alanine, and glycine, levels of which were notably elevated in inoculated plants (Shojima et al. 1990; Kuzmicheva et al. 2017). Enhanced iron acquisition not only stimulates the phenylpropanoid pathway, boosting the activity of enzymes responsible for phenolic compound biosynthesis, but also reinforces the plant’s defense mechanisms and stress resilience. Furthermore, improved iron homeostasis driven by siderophore release triggers plant nitrate assimilation and growth (Araki et al. 2015). These findings align with previous laboratory studies, where inoculation with *P. oryzihabitans* RL18 induced a metabolic shift in *Salicornia europaea* towards amino acid biosynthesis (Ferreira et al. 2023). Together, these results suggest that the application of PGPB can fundamentally reprogram plant metabolism, enhancing both growth and stress tolerance under saline conditions.

In addition, inoculated plants exhibited increased sulfur accumulation and greater resistance to stress, likely linked to the biosynthesis of sulfur-containing metabolites like cysteine, glutathione, and methionine (Li et al. 2020). These compounds, as well as the phenolic compounds produced by these plants, greatly enhance their resistance to diverse stresses (Mandal et al. 2022).

Inoculated plants accumulated low-stress-indicator compounds like unsaturated fatty acids and sterols, which may be correlated with the upregulation of malonate and mevalonate pathways. This suggests enhanced stress resilience, though further research is needed. Unsaturated fatty acids stabilize cellular structures and influence membrane fluidity and signaling (He and Ding 2020). Sterols maintain membrane integrity under stress (Du et al. 2022). Additionally, inoculation decreased oxalic acid concentration, potentially enhancing nutritional value and safety by reducing kidney stone risk in consumers (Sc Noonan 1999).

It is known that PGPB primes plants for improved synthesis of bioactive compounds with antioxidant and antimicrobial properties, contributing to enhanced stress tolerance and disease resistance. PGPB, such as *Bacillus subtilis* and *Pseudomonas fluorescens*, have been reported to upregulate genes like *DAHPS* (3-deoxy-D-arabino-heptulosonate 7-phosphate synthase) and *PAL* (Phenylalanine Ammonia-Lyase), which are critical for phenolic acid and flavonoid production (Galicia-Campos et al. 2022). For example, inoculation with *Azospirillum brasilense* increased the activity of enzymes in the phenylpropanoid pathway, leading to higher accumulation of caffeic and ferulic acids in maize (Aguiar et al. 2018). Similarly, rhizobacteria-induced expression of flavonoid biosynthesis genes such as *CHS* (Chalcone Synthase) and *CHI* (Chalcone Isomerase) has been observed in legumes, resulting in enhanced flavonoid production (Galicia-Campos et al. 2022). Additionally, PGPB like *Burkholderia phytofirmans PsJN* have been shown to boost anthocyanin levels in grape plants by upregulating flavonoid-related genes (Ngalimat et al. 2021). Beyond phenolic compounds, certain PGPB can also influence fatty acid profiles, as seen with *Pseudomonas putida* in *Arabidopsis*, potentially enhancing plant defense mechanisms (Ngalimat et al. 2021). These effects are often mediated through hormonal signaling pathways, such as jasmonic acid and ethylene, or through activation of transcription factors like MYB and bHLH, which regulate secondary metabolite biosynthesis (Galicia-Campos et al. 2022).

In the pilot-scale setup, non-inoculated plants accumulated higher levels of carboxylic acids than inoculated plants. This may be due to PGPB-induced metabolic reprogramming, which redirects carbon flux away from carboxylic acid synthesis toward the production of metabolites such as phenolic compounds and flavonoids that enhance stress tolerance (Badri and Vivanco 2008). Additionally, non-inoculated plants might accumulate carboxylic acids as a stress response, using these compounds to regulate pH and serve as osmoprotectants under adverse conditions (Wang et al. 2024)(Fougère et al. 1991). In contrast, the presence of beneficial bacteria improves nutrient uptake and overall plant health, thereby reducing the need for such compensatory mechanisms (Mokrani et al. 2020).

To further elucidate our findings, our experimental design included both microcosm and pilot-scale systems. The microcosm experiments, performed under tightly controlled conditions, allowed us to clearly observe the metabolic shifts induced by PGPB inoculation. Conversely, the pilot-scale systems—subject to greater environmental variability, including fluctuations in oxygenation, temperature, and light intensity—better reflect real-world conditions, where both biotic and abiotic elicitors interact to modulate plant metabolic responses (Petrova et al. 2024).

While it remains unclear whether these metabolic shifts directly result from inoculation or are a consequence of phytoextraction, we hypothesize that inoculation induces metabolic changes that enhance nutrient uptake and alter the phytochemical composition of the plant, providing a valuable direction for future research.

### Enhancing the market value of cultivated *Salicornia europaea* through biostimulation with PGPB

In aquaponic conditions, the cultivation of edible halophytes like *S. europaea* offers a dual benefit of nutrient removal from aquaculture water and increased productivity (Marques et al. 2021), diversifying the range of products that can be harvested from aquaculture systems (Turcios and Papenbrock 2014). The inoculation with PGPB led to an enrichment in specific bioactive compounds in *S. europaea*, such as eicosanol, docosanol, luteolin, taxifolin, formononetin, gallocatechin, kaempferol, quercetin, and acacetin, which possess pharmacological value including antimicrobial, antitumoral, and antioxidant activities (Faraji and Lindsay 2004; Mukherjee et al. 2013; Montserrat-De La Paz et al. 2014; Wang et al. 2018; Akram et al. 2020; Singh et al. 2020; Yang et al. 2020). Additionally, certain compounds such as medioresinol and norbellidifodin, found only in plants under the influence of *P. oryzihabitans* RL18, have shown antimicrobial potential (Hwang et al. 2012). Notably, inoculated plants synthesized borneol, facilitating drug delivery to the central nervous system, and tocopherol, enhancing their antioxidant potential (Zhang et al. 2017; Szewczyk et al. 2021).

The enhanced secondary metabolite profile observed in inoculated *Salicornia europaea* holds significant economic promise. The increased levels of antioxidants, phenolic acids, and flavonoids suggest that this halophyte could be developed into high-value products for the food and pharmaceutical industries. For example, the enriched bioactive compounds may serve as natural preservatives or functional ingredients in nutraceuticals, while their antioxidant and antimicrobial properties could be harnessed in novel therapeutic formulations. Highlighting these potential commercialization pathways not only underscores the practical relevance of our research but also demonstrates a clear route to adding value to sustainable saline agriculture.

These findings underscore the potential of PGPB-assisted cultivation of *Salicornia europaea* to enhance its nutraceutical value and expand its pharmacological interest, offering promising applications in aquaponic systems for nutrient removal and diversified product harvests.

## Conclusion

This study underscores the potential of PGPB inoculation to enhance the metabolite profile of *Salicornia europaea* in aquaponic systems, while offering a transformative vision for large-scale aquaponics. The significant metabolic shifts induced by the co-inoculation of *Brevibacterium casei* EB3 and *Pseudomonas oryzihabitans* RL18, particularly the accumulation of bioactive compounds, such as phenolic acids, flavonoids, and fatty acids, with antioxidant and antimicrobial properties, suggest that integrating these bacterial inoculants into commercial aquaponic operations could dramatically boost productivity and sustainability.

In essence, our findings pave the way for aquaponic systems that not only maximize resource recycling and crop yield but also deliver high-value, nutraceutical and pharmaceutical ingredients. This breakthrough approach holds the potential to revolutionize sustainable food production on a commercial scale, aligning with global efforts to mitigate climate change and promote environmental resilience.

## Electronic supplementary material

Below is the link to the electronic supplementary material.

Supplementary Material 1

## Data Availability

No datasets were generated or analysed during the current study.
