# Supplementary material for: PGPB-driven bioenrichment and metabolic modulation of Salicornia europaea under marine Aquaponic conditions
Source: World J Microbiol Biotechnol. 2025 Apr 7;41(4):124. doi: 10.1007/s11274-025-04335-5 (PMC11972990; doi:10.1007/s11274-025-04335-5)
Supplement: Supplementary file 1 — Supplementary Material 1 [file 11274_2025_4335_MOESM1_ESM.docx]

**Bioenrichment and Metabolic Modulation of *Salicornia europaea* in Marine Aquaponic Systems**

Maria J. Ferreira^1,2,3^, Erika Garcia-Cardesín^3^, I. Natalia Sierra-Garcia^1^, Diana C. G. A. Pinto^2^, Javier Cremades^3^, Helena Silva^1^ and Ângela Cunha^1^

^1^ Department of Biology & Center for Environmental and Marine Studies (CESAM), University of Aveiro, Campus de Santiago, 3810-193 Aveiro, Portugal.

^2^ LAQV-REQUIMTE & Department of Chemistry, University of Aveiro, Campus de Santiago, 3810-193 Aveiro, Portugal.

^3^ Interdisciplinary Center for Chemistry and Biology (CICA), University of A Coruña, 15071 A Coruña, Spain.

**Corresponding author email:** [mjoaovf@ua.pt](mailto:mjoaovf@ua.pt)

**Supplementary Figures**

| 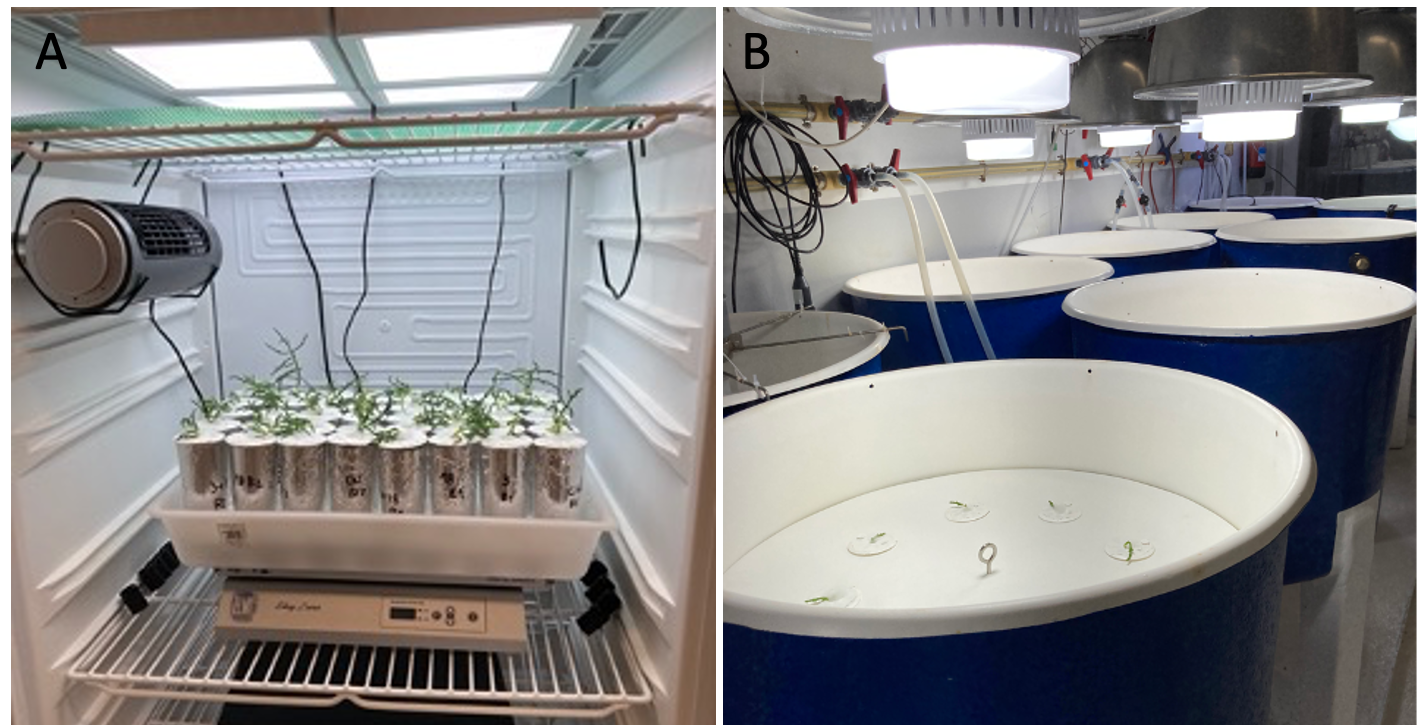 |
| --- |

Supplementary Figure S1. Aquaponic cultivation of *Salicornia europaea* exposed to different inoculants under controlled conditions inside a growth chamber (A) and in an indoor pilot-scale system. (B).

Supplementary Figure S2. Detailed phytochemical profile of plants grown in aquaponic microcosm conditions, according to compounds detected by UHPLC-MS (A-Phenolic acids; B- Flavonoids). NI - non-inoculated plants; EB3 – *Brevibacterium* *casei* EB3; RL18 - *Pseudomonas* *oryzihabitans* RL18; EB3+RL18 – *Brevibacterium* *casei* EB3 + *Pseudomonas* *oryzihabitans* RL18. Columns indicate a mean of 3 biological replicates, and bars standard error Data were compared by Kruskal-Wallis test . Different letter (a-b) mean significant differences.


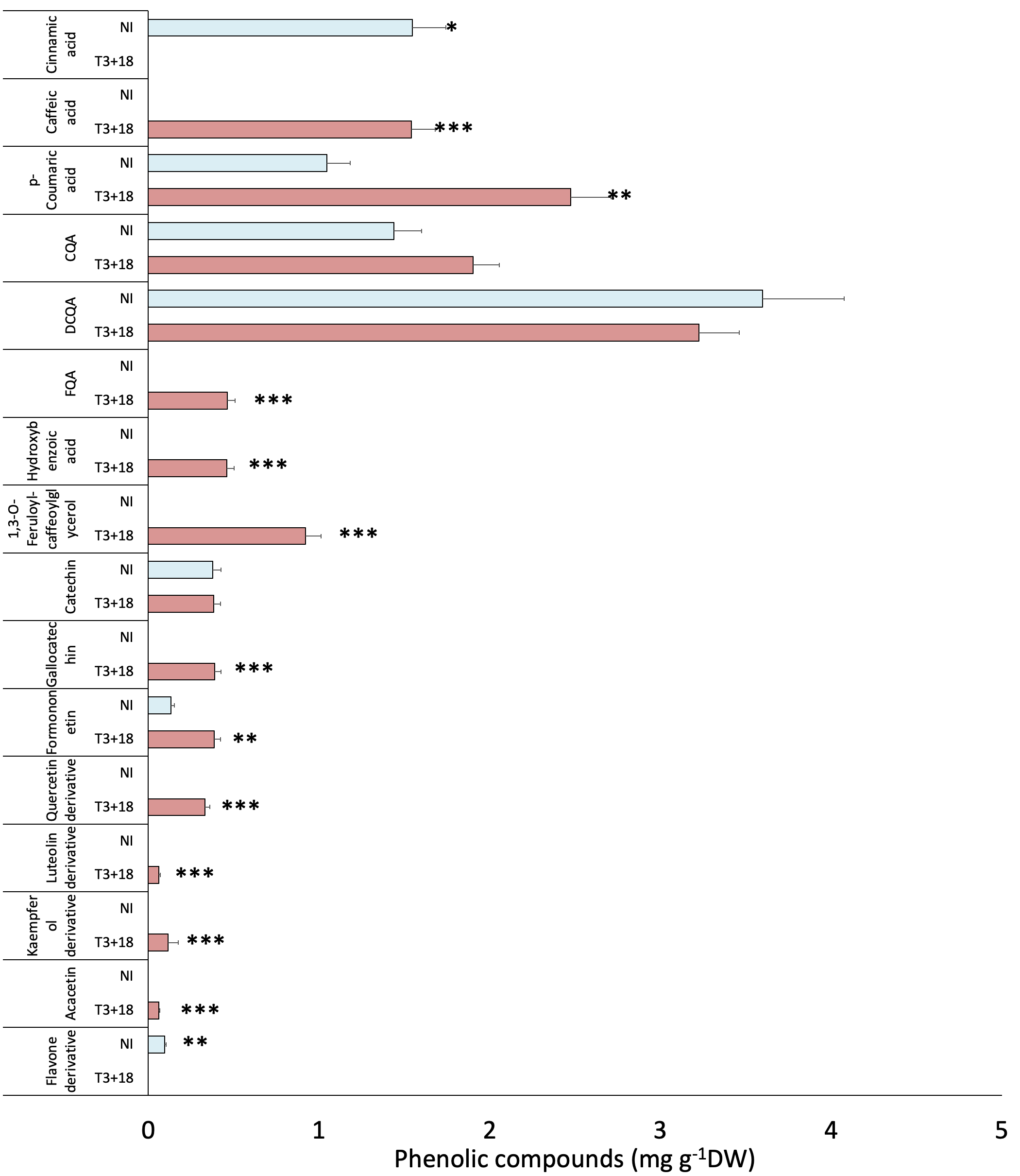


Supplementary Figure S3. Detailed phytochemical profile of plants grown in aquaponic pilot-scale conditions, according to compounds detected by UHPLC-MS. NI- non-inoculated plants; EB3+RL18 – inoculated plants with *Brevibacterium casei* EB3 and *Pseudomonas oryzihabitans* RL18. Columns indicate a mean of 3 replicates, and bars standard error Data were compared by Mann-Whitney U test . Significant differences: *p<0.05; ** p<0.005; ***p<0.001.

**Supplementary Tables**

Supplementary Table S1. Summary of the plant growth promotion activities and salt tolerance of the tested bacterial strains (Ferreira et al. 2021)


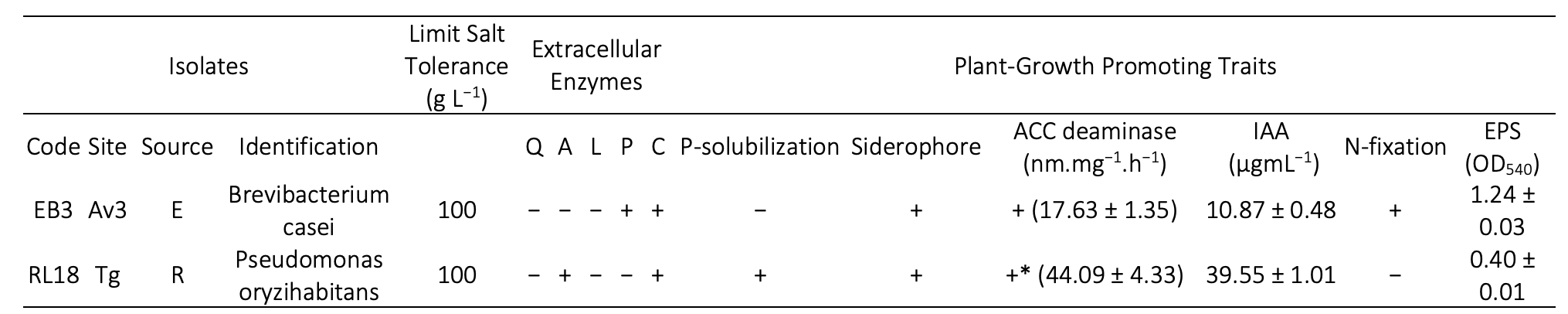


E—Endosphere; R—Rhizosphere; Q—Chitinase; A—Amylase; L—Lipase; P—Protease; C—Cellulase; Av3 - Boco (Aveiro); Tg – Tagus Estuary (Lisbon); + positive; − negative; +* visible growth on solid DF +ACC medium.

Supplementary Table S2. Phytochemical profile of plants grown in aquaponic microcosm and pilot-scale conditions, according to chemical families of compounds detected by GC-MS and UHPLC-MS (mg g ^-1^DW).

|  | Microcosm | | | | Pilot-scale | |
| --- | --- | --- | --- | --- | --- | --- |
| Compounds | NI | EB3 | RL18 | EB3+RL18 | NI | EB3+RL18 |
| Amino acids | 18.6±3.7^a^ | 14.4±2.0^a^ | 13.8±1.9^a^ | 16.8±3.5^a^ | 9.9±3.5 | 12.7±2.3 |
| Sugar acids | 15.6±1.3^a^ | 14.9±0.5^a^ | 14.2±1.0^a^ | 13.3±1.7^a^ | 7.1±1.5 | 5.2±1.2 |
| Sugars | 61.6±3.3^a^ | 63.3±4.6^a,b^ | 72.1±3.0^b^ | 72.0±3.2^b^ | 51.7±2.6 | 54.4±2.3 |
| SFA | 23.0±2.3^a^ | 15.81.1^a^ | 20.2±2.1^a^ | 24.7±2.4^a^ | 16.0±3.5 | 21.7±1.8 |
| UFA | 17.1±2.2^a^ | 18.4±0.8^a^ | 17.8±2.3^a^ | 23.2±3.3^a^ | 11.1±1.6 | 19.0±2.1 |
| Monoacylglycerols | 15.0±2.6^a^ | 16.7±1.4^a^ | 19.8±4.5^a^ | 11.8±1.6^a^ | 12.6±1.9 | 18.5±3.0 |
| Carboxylic acids | 5.3±0.5^a^ | 6.9±0.6^b^ | 6.1±0.5^b^ | 5.5±0.5^a,b^ | 5.3±0.4^**^ | 3.2±0.2 |
| Sugar alcohols | 2.9±0.3^a^ | 1.7±0.3^a^ | 2.0±0.1^a^ | 2.2±0.3^a^ | 1.0±0.1 | 1.0±0.1 |
| Alcohols | 3.4±0.4^a^ | 6.2±0.4^a,b^ | 6.6±0.3^b^ | 4.0±0.7^a^ | 2.4±0.2 | 3.8±0.2^**^ |
| Sterols | 4.4±0.4^a^ | 3.8±0.5^a^ | 5.3±0.5^a^ | 5.1±0.8^a^ | 5.7±0.2 | 5.6±0.6 |
| Phenolic acids | 11.2±1.5^a^ | 11.1±1.5^a^ | 10.7±0.9^a^ | 12.5±0.4^a^ | 8.1±1.0 | 10.1±0.8 |
| Cinnamic acid derivatives | 3.7±0.6^a^ | 4.2±0.6^a^ | 2.7±0.3^a^ | 3.4±0.1^a^ | 2.6±0.3 | 4.0±0.4^*^ |
| Chlorogenic acids | 7.5±0.9^a^ | 6.9±0.9^a^ | 8.0±0.7^a^ | 9.1±0.3^a^ | 5.0±0.6 | 5.6±0.4 |
| Hydroxybenzoic acid | 0.0±0.0 | 0.0±0.0 | 0.0±0.0 | 0.0±0.0 | 0.0±0.0 | 0.5±0.0^***^ |
| Flavonoids | 1.5±0.2^a^ | 2.0±0.3^a^ | 1.7±0.2^a^ | 1.9±0.0^a^ | 0.6±0.1 | 1.7±0.2^**^ |
| Flavones | 0.1±0.0^a^ | 0.2±0.0^b^ | 0.2±0.0^b^ | 0.3±0.0^b^ | 0.0±0.0 | 0.0±0.0 |
| Flavonols | 0.4±0.1^a^ | 0.2±0.0^a^ | 0.2±0.0^a^ | 0.2±0.0^a^ | 0.0±0.0 | 0.5±0.1^**^ |
| Flavanols | 0.5±0.1^a^ | 0.7±0.1^a^ | 0.7±0.1^a^ | 0.7±0.0^a^ | 0.4±0.0 | 0.8±0.1^*^ |
| Isoflavones | 0.3±0.0^a^ | 0.4±0.1^a^ | 0.3±0.0^a^ | 0.5±0.0^b^ | 0.1±0.0 | 0.4±0.0^**^ |
| Flavanonols | 0.0±0.0^a^ | 0.2±0.0^b^ | 0.2±0.0^b^ | 0.0±0.0^a^ | 0.0±0.0 | 0.0±0.0 |
| Other flavonoids | 0.2±0.0^a^ | 0.2±0.0^a^ | 0.2±0.0^a^ | 0.2±0.0^a^ | 0.0±0.0 | 0.0±0.0 |

NI- non-inoculated plants; EB3 – plants inoculated with *Brevibacterium casei* EB3; RL18 – plants inoculated with *Pseudomonas oryzihabitans* RL18; EB3+RL18 –plants inoculated with *Brevibacterium casei* EB3 and *Pseudomonas oryzihabitans* RL18.

Values represent the mean of 3 biological replicates ± standard error. Values with different letter (a-b, *p* < 0.05) or * (*p* < 0.05), ** (*p* < 0.005) and *** (*p* < 0.001) along the row indicate significant statistical differences between treatments

Supplementary Table S3. Compounds identified by GC-MS in *S. europaea* grown in aquaponic microcosm conditions, in non-inoculated plants (NI), and in plants inoculated with *Brevibacterium* *casei* EB3 (EB3), *Pseudomonas* *oryzihabitans* RL18 (RL18) or a mixture of *Brevibacterium* *casei* EB3 and *Pseudomonas* *oryzihabitans* (EB3+RL18).

| Identification ^a^ | NI | EB3 | RL18 | EB3+RL18 |
| --- | --- | --- | --- | --- |
| ***Amino acids*** |  |  |  |  |
| Glycine | 13.2±2.76 ^a^ | 11.5±1.63 ^a^ | 10.9±1.72 ^a^ | 12.2±2.66 ^a^ |
| Alanine | 3.6±1.14 ^a^ | 0.9±0.90 ^a^ | 0.0±0.00 ^a^ | 1.7±1.08 ^a^ |
| Valine | 1.2±0.15 ^a^ | 1.1±0.04 ^a^ | 0.8±0.31 ^a^ | 0.7±0.23 ^a^ |
| Leucine | 0.3±0.21 ^a^ | 0.8±0.28 ^a^ | 0.2±0.16 ^a^ | 0.6±0.19 ^a^ |
| Iso-leucine | 0.3±0.21 ^a^ | 0.2±0.16 ^a^ | 0.5±0.20 ^a^ | 0.6±0.20 ^a^ |
| Threonine | - | - | nq | nq |
| Glutamic acid | - | nq | nq | nq |
| Asparagine | - | nq | - | - |
| Tyramine | - | - | nq | - |
| Proline | - | - | - | nq |
| Lysine | - | - | nq | - |
| ***Carboxylic acids*** |  |  |  |  |
| Lactic acid | 0.6±0.13 ^a^ | 0.6±0.01 ^a^ | 0.6±0.01 ^a^ | 0.7±0.10 ^a^ |
| Oxalic acid | 1.2±0.13 ^a^ | 1.8±0.20 ^a^ | 1.7±0.15 ^a^ | 0.9±0.24 ^a^ |
| Succinic acid | 1.4±0.23^a^ | 0.8±0.10 ^b^ | 0.4±0.14 ^b^ | 0.6±0.01^b^ |
| Malic acid | 1.1±0.10 ^a^ | 1.0±0.13 ^a^ | 1.0±0.13 ^a^ | 1.0±0.15 ^a^ |
| Fumaric acid | 0.3±0.13 ^a^ | 0.3±0.13 ^a^ | 0.1±0.10 ^a^ | 0.2±0.12 ^a^ |
| Propanoic acid | 0.5±0.18 ^a^ | 0.3±0.13 ^a^ | 0.7±0.09 ^a^ | 0.6±0.05 ^a^ |
| Acetic acid | 0.4±0.16 ^a^ | 0.8±0.15 ^a^ | 0.8±0.03 ^a^ | 1.0±0.11 ^a^ |
| Glycolic acid | 0.6±0.19 ^a^ | 0.1±0.10 ^a^ | 0.0±0.00 ^a^ | 0.0±0.00 ^a^ |
| Citric acid | 0.0±0.00 ^a^ | 0.0±0.00 ^a^ | 0.0±0.00 ^a^ | 0.2±0.12 ^a^ |
| ***Sugar acids*** |  |  |  |  |
| Glyceric acid | 0.8±0.01 ^a^ | 0.9±0.10 ^a^ | 0.7±0.01 ^a^ | 0.7±0.01 ^a^ |
| Threonic acid | 1.3±0.03 ^a^ | 1.6±0.20 ^a^ | 1.5±0.15 ^a^ | 1.3±0.12 ^a^ |
| Arabinonic acid | 1.2±0.01 ^a^ | 1.3±0.09 ^a^ | 1.5±0.25 ^a^ | 0.6±0.15 ^b^ |
| Ribonic acid | 2.8±0.38 ^a^ | 3.5±0.41 ^a^ | 2.5±0.19 ^a^ | 2.5±0.19 ^a^ |
| Glutaric acid | 0.6±0.50 ^a^ | 0.0±0.00 ^a^ | 0.1±0.11 ^a^ | 0.4±0.13 ^a^ |
| Hexonic acid | 1.2±0.03 ^a^ | 0.6±0.20 ^a^ | 1.0±0.03 ^a^ | 0.6±0.19 ^a^ |
| Glucaric acid | 3.5±1.13 ^a^ | 2.4±0.58 ^a^ | 2.7±0.49 ^a^ | 1.5±0.53 ^a^ |
| Gluconic acid | 1.0±0.31 ^a^ | 0.7±0.28 ^a^ | 0.1±0.10 ^a^ | 0.5±0.18 ^a^ |
| Glucoronic acid | 0.6±0.24 ^a^ | 0.6±0.24 ^a^ | 0.6±0.15 ^a^ | 0.9±0.31 ^a^ |
| Galactaric acid | 0.4±0.19 ^a^ | 0.6±0.58 ^a^ | 1.0±0.55 ^a^ | 1.7±0.58 ^a^ |
| Tartaric acid | 0.4±0.26 ^a^ | 1.6±0.25 ^a^ | 1.4±0.21 ^a^ | 1.2±0.41 ^a^ |
| Galactonic acid | 0.0±0.00 ^b^ | 0.2±0.14 ^a,b^ | 0.4±0.14 ^a,b^ | 0.9±0.12 ^a^ |
| Mannonic acid | 0.0±0.00 ^b^ | 0.6±0.15 ^a^ | 0.5±0.10 ^a^ | 0.0±0.00 ^b^ |
| Galacturonic acid | 0.0±0.00 ^a^ | 0.1±0.10 ^a^ | 0.0±0.00 ^a^ | 0.1±0.09 ^a^ |
| Gulonic acid | 0.0±0.00 ^a^ | 0.0±0.00 ^a^ | 0.2±0.11 ^a^ | 0.2±0.12 ^a^ |
| Xylonic acid | 0.0±0.00 ^a^ | 0.0±0.00 ^a^ | 0.0±0.00 ^a^ | 0.4±0.18 ^a^ |
| ***Sugar alcohols*** |  |  |  |  |
| Glycerol | 2.1±0.25^a^ | 1.1±0.22 ^a,b^ | 2.0±0.59 ^a,b^ | 1.5±0.26 ^b^ |
| Erythritol | 0.1±0.02 ^a^ | 0.1±0.03 ^a^ | 0.1±0.01 ^a^ | 0.1±0.02 ^a^ |
| Xylitol | 2.1±0.25 ^a^ | 1.1±0.22 ^a^ | 2.0±0.59 ^a^ | 1.5±0.26 ^a^ |
| Mannitol | 0.1±0.03 ^a^ | 0.1±0.02 ^a^ | 0.0±0.02 ^a^ | 0.0±0.02 ^a^ |
| Arabinol | 0.0±0.01 ^a^ | 0.0±0.00 ^a^ | 0.0±0.01 ^a^ | 0.0±0.01 ^a^ |
| Myo-inositol | 0.5±0.04 ^a^ | 0.3±0.08 ^a^ | 0.4±0.03 ^a^ | 0.4±0.08 ^a^ |
| Ribitol | 0.0±0.00^a^ | 0.0±0.01^a^ | 0.0±0.01 ^a^ | 0.0±0.00^a^ |
| Inositol | 0.0±0.00 ^a^ | 0.0±0.01 ^a^ | 0.0±0.00 ^a^ | 0.0±0.00 ^a^ |
| Fucitol | 0.0±0.00 ^a^ | 0.0±0.01 ^a^ | 0.0±0.00 ^a^ | 0.0±0.00 ^a^ |
| Dulcitol | 0.0±0.00 ^a^ | 0.0±0.00 ^a^ | 0.0±0.01 ^a^ | 0.0±0.01 ^a^ |
| ***Alcohols*** |  |  |  |  |
| Phytol | 0.3±0.03 ^a^ | 0.3±0.01 ^a^ | 0.4±0.05 ^a^ | 0.3±0.01 ^a^ |
| 1-Eicosanol | 0.3±0.06 ^b^ | 0.8±0.12 ^a ,b^ | 0.8±0.03 ^a^ | 0.3±0.10 ^b^ |
| Docosanol | 1.1±0.13 ^b^ | 2.6±0.25 ^a^ | 2.7±0.14 ^a^ | 1.3±0.35 ^b^ |
| 1-Hexatriacontanol | 0.9±0.08 ^a^ | 0.8±0.30 ^a^ | 0.7±0.29 ^a^ | 0.4±0.19 ^a^ |
| 1-Hexacosanol | 0.2±0.07 ^a^ | 0.2±0.07 ^a^ | 0.2±0.05 ^a^ | 0.2±0.06 ^a^ |
| 1-Octacosanol | 0.4±0.04 ^a^ | 0.4±0.03 ^a^ | 0.4±0.01 ^a^ | 0.4±0.03 ^a^ |
| 1-Triacontanol | 0.2±0.06 ^a,b^ | 0.0±0.04 ^b^ | 0.1±0.05 ^a,b^ | 0.3±0.01^a^ |
| 1,2,4-Butanetriol | 0.1±0.05 ^a^ | 0.3±0.13 ^a^ | 0.2±0.07 ^a^ | 0.3±0.10 ^a^ |
| Tricosanol | 0.1±0.05 ^a^ | 0.2±0.07 ^a^ | 0.1±0.06 ^a^ | 0.1±0.05 ^a^ |
| 11-Bromo-1-undecanol | 0.0±0.00 ^a^ | 0.0±0.04 ^a^ | 0.0±0.00 ^a^ | 0.0±0.00 ^a^ |
| Tetracosanol | 0.0±0.00 ^a^ | 0.0±0.04 ^a^ | 0.0±0.00 ^a^ | 0.0±0.00 ^a^ |
| 1-Heneicosanol | 0.0±0.00 ^a^ | 0.1±0.05 ^a^ | 0.2±0.04 ^a^ | 0.1±0.05 ^a^ |
| 1,11-Undecanediol | 0.0±0.00 ^a^ | 0.0±0.04 ^a^ | 0.0±0.00 ^a^ | 0.0±0.00 ^a^ |
| 1-Decanol | 0.0±0.00 ^a^ | 0.0±0.00 ^a^ | 0.1±0.06 ^a^ | 0.0±0.04 ^a^ |
| ***Sugars*** |  |  |  |  |
| Arabinose | 0.0±0.00 ^b^ | 2.4±0.87 ^a,b^ | 4.1±1.09 ^a^ | 2.3±1.18 ^a,b^ |
| Ribose | 8.8±1.15 ^b^ | 9.9±1.36 ^b^ | 10.8±2.17 ^a^ | 8.4±1.46 ^b^ |
| Galactose | 22.6±2.78 ^a^ | 12.9±1.61 ^a^ | 16.9±2.73 ^a^ | 14.8±1.34 ^a^ |
| Glucose | 14.9±1.74 ^a^ | 15.3±2.60 ^a^ | 15.0±1.99 ^a^ | 19.8±2.02 ^a^ |
| Psicose | 6.5±0.52 ^a^ | 4.5±0.77 ^a^ | 0.8±0.48 ^a^ | 4.3±0.76 ^a^ |
| Fructose | 0.0±0.00 ^a^ | 1.6±1.00 ^a^ | 2.3±1.07 ^a^ | 1.6±1.14 ^a^ |
| Allose | 1.2±0.52 ^a^ | 3.6±1.20 ^a^ | 3.4±0.83 ^a^ | 1.9±0.73 ^a^ |
| Tagatose | 1.5±0.60 ^a^ | 3.6±1.49 ^a^ | 2.7±0.69 ^a^ | 3.1±0.97 ^a^ |
| Fucose | 0.0±0.00 ^b^ | 1.6±0.51^b^ | 1.1±0.73^b^ | 4.6±1.03 ^a^ |
| Mannose | 1.2±0.56 ^a^ | 5.2±1.30 ^a^ | 8.7±2.06 ^a^ | 4.6±1.83 ^a^ |
| Erythrose | 0.0±0.00 ^a^ | 0.4±0.41 ^a^ | 0.0±0.00 ^a^ | 0.0±0.00 ^a^ |
| Sucrose | 2.8±0.40 ^a,b^ | 2.0±0.75 ^a,b^ | 1.5±0.46 ^b^ | 4.2±0.92 ^a^ |
| Xylose | 3.1±0.46 ^a^ | 0.0±0.00^b^ | 0.0±0.00 ^b^ | 0.0±0.00 ^b^ |
| ***Saturated fatty acids*** |  |  |  |  |
| Butanoic acid | 0.0±0.00 ^b^ | 2.9±0.91^a^ | 1.0±0.45 ^a,b^ | 1.5±0.50 ^a,b^ |
| Lauric acid | 2.2±0.30 ^a,b^ | 0.8±0.49 ^b^ | 1.6±0.50 ^a,b^ | 2.7±0.34 ^a^ |
| Myristic acid | 2.3±0.04 ^a^ | 1.9±0.38 ^a^ | 1.8±0.36 ^a^ | 1.5±0.47 ^a^ |
| Palmitic Acid | 11.3±0.92 | 6.6±0.40 ^a^ | 10.2±0.87 ^a^ | 11.8±1.10 ^a^ |
| Stearic acid | 4.6±1.36 ^a,b^ | 1.9±0.20 ^b^ | 2.4±0.77 ^b^ | 4.1±0.67 ^a^ |
| Behenic acid | 1.0±0.68 ^a,b^ | 0.0±0.00 ^b^ | 0.0±0.00 ^b^ | 1.9±0.39 ^a^ |
| Arcachidic acid | 0.4±0.43 ^a^ | 0.0±0.00 ^a^ | 1.3±0.73 ^a^ | 0.9±0.54 ^a^ |
| Lignoceric acid | 1.3±0.56 ^a^ | 2.2±0.59 ^a^ | 1.4±0.45 ^a^ | 2.3±0.03 ^a^ |
| ***Unsaturated fatty acids*** | |  |  |  |
| Oleic acid | 4.1±1.54 ^a^ | 3.4±1.06 ^a^ | 7.1±1.07 ^a^ | 8.1±1.60 ^a^ |
| Linoleic acid | 7.8±0.67 ^a^ | 7.8±1.11 ^a^ | 5.1±0.12 ^a^ | 8.9±1.19 ^a^ |
| α-linolenic acid | 5.2±0.14 ^a^ | 4.3±0.03 ^a^ | 4.2±0.10 ^a^ | 5.2±0.86 ^a^ |
| Eicos-11-enoic acid | 0.8±0.83 ^b^ | 2.2±0.97 ^a^ | 0.0±0.00^b^ | 0.0±0.00^b^ |
| 2-Hexenedioic acid | 0.0±0.00 ^a^ | 0.0±0.00 ^a^ | 0.8±0.78 ^a^ | 0.0±0.00 ^a^ |
| 2-Pentenoic acid | 0.0±0.00 ^a^ | 0.0±0.00 ^a^ | 0.8±0.78 ^a^ | 0.0±0.00 ^a^ |
| trans-2-Hexadecenoic acid | 0.0±0.00 ^a^ | 0.0±0.00 ^a^ | 0.0±0.00 ^a^ | 0.8±0.79 ^a^ |
| ***Monoacylglycerols*** |  |  |  |  |
| Monopalmitin | 8.5±1.13 ^a^ | 8.7±1.09 ^a^ | 10.9±2.32 ^a^ | 7.3±0.84 ^a^ |
| Monostearin | 3.4±1.13 ^a,b^ | 6.4±0.16 ^a^ | 5.2±2.32 ^a,b^ | 1.9±0.83 ^b^ |
| Monoarachidin | 1.8±0.90 ^a^ | 0.9±0.55 ^a^ | 1.8±0.85 ^a^ | 1.7±0.55 ^a a^ |
| Monomyristin | 0.4±0.37 ^a^ | 1.5±0.46 ^a^ | 1.1±0.47 ^a^ | 1.7±0.89 |
| ***Sterols*** |  |  |  |  |
| Stigmasterol | 1.4±0.06 ^a^ | 1.6±0.24 ^a^ | 1.4±0.04 ^a^ | 1.4±0.05 ^a^ |
| β.sitosterol | 1.7±0.07 ^a^ | 1.5±0.05 ^a^ | 1.8±0.25 ^a^ | 1.6±0.07 ^a^ |
| Stigmastanol | 0.0±0.00 ^a^ | 0.2±0.22 ^a^ | 0.6±0.28 ^a^ | 0.8±0.40 ^a^ |
| Ergost-5-ene-3,25-diol | 0.2±0.22 ^b^ | 0.7±0.29 ^a,b^ | 0.4±0.28 ^b^ | 1.3±0.32 ^a^ |
| Cholest-5-en-3-ol, (3.alpha.) | 0.7±0.30^a^ | 0.0±0.00 ^b^ | 0.0±0.00 ^b^ | 0.0±0.00 ^b^ |
| Gorgosterol | 0.0±0.00 ^b^ | 0.0±0.00 ^b^ | 0.9±0.43 ^a^ | 0.0±0.00 ^b^ |
| ***Terpenoids*** |  |  |  |  |
| Neophytadiene | nq | nq | nq | nq |
| Thymol | - | - | nq | - |
| Borneol | - | nq | nq | nq |
| ***Other compounds*** |  |  |  |  |
| Niacin | nq | nq | nq | - |
| Uridine | nq | nq | nq | nq |
| Diacetone alcohol | nq | nq | nq | nq |
| Tocopherol | - | - | - | nq |
| Glyceryl-glycoside | nq | nq | nq | nq |

^a^ All compounds possessing hydroxy groups are identified as the correspondent TMS derivatives. Compounds were identified by comparison with the GC-MS spectral libraries NIST14.lib and WILEY229.lib. ( -) - not detected; (+)- detected; (nq)- not quantified due to the lack of an appropriate standard. Values with different letter (a-c) along the row indicates significant statistical differences between treatments (One-way ANOVA with Tukey's multiple comparisons post hoc test or Kruskal-Wallis test, p<0.05)

Supplementary Table S4. Compounds identified by GC-MS on *S. europaea* samples, non-inoculated plants (NI) and inoculated plants (EB3+RL18-*Brevibacterium* *casei* EB3 and *Pseudomonas* *oryzihabitans*) under aquaponic pilot-scale conditions.

| Identification ^a^ | NI | EB3+RL18 |
| --- | --- | --- |
| ***Amino acids*** |  |  |
| Glycine | 8.0±2.95 | 10.9±2.29 |
| Alanine | 0.0±0.00 | 0.9±0.85 |
| Valine | 0.0±0.00 | 0.9±0.85 |
| Leucine | 0.6±0.19 | 0.1±0.14 |
| Isoleucine | 0.±0.20 | 0.3±0.19 |
| Threonine | - | + |
| Asparagine | - | + |
| ***Carboxylic acids*** |  |  |
| Lactic Acid | 0.7±0.09* | 0.6±0.01 |
| Oxalic acid | 0.6±0.22 | 0.0±0.00 |
| Succinic acid | 0.6±0.01 | 0.4±0.12 |
| Malic acid | 1.1±0.11 | 0.8±0.20 |
| Fumaric acid | 1.1±0.11 | 0.8±0.20 |
| Propanoic acid | 0.9±0.25* | 0.5±0.09 |
| Acetic acid | 0.8±0.12* | 0.7±0.09 |
| Caproic | 0.5±0.10* | 0.2±0.12 |
| Citric | 0.1±0.09 | 0.0±0.00 |
| ***Sugar acids*** |  |  |
| Glyceric acid | 0.9±0.20 | 0.5±0.10 |
| Threonic acid | 1.1±0.18 | 1.0±0.23 |
| Arabinonic acid | 0.5±0.18 | 0.6±0.14 |
| Ribonic acid | 1.2±0.34 | 1.2±0.37 |
| Glutaric acid | 0.1±0.10 | 0.1±0.10 |
| hexonic acid | 0.7±0.05 | 0.4±0.14 |
| Glucaric acid | 1.1±0.26 | 0.4±0.14 |
| Gluconic acid | 0.1±0.09 | 0.0±0.00 |
| Glucuronic acid | 0.4±0.20 | 0.0±0.00 |
| Galactaric acid | 0.0±0.00 | 0.1±0.10 |
| Tartaric acid | 0.4±0.14 | 0.4±0.12 |
| Galactonic acid | 0.4±0.23 | 0.4±0.28 |
| Mannonic acid | 0.1±0.10 | 0.0±0.00 |
| Galacturonic acid | 0.1±0.10 | 0.0±0.00 |
| Gulonic acid | 0.4±0.13* | 0.0±0.00 |
| Xylonic acid | 0.4±0.13 | 0.0±0,00 |
| **Sugar alcohols** |  |  |
| Glycerol | 0.6±0.04 | 0.6±0.07 |
| Erythrytol | 0.0±0.01 | 0.1±0.01 |
| Xylitol | 0.0±0.01 | 0.0±0.01 |
| D-Mannitol | 0.1±0.02 | 0.1±0.01 |
| L-(-)-Arabitol | 0.1±0.04 | 0.0±0.01 |
| Myo-Inositol | 0.1±0.04 | 0.2±0.01 |
| Ribitol | 0.0±0.01 | 0.0±0.01 |
| ***Alcohols*** |  |  |
| Phytol | 0.4±0.08 | 03±0.06 |
| 1-Eicosanol | 0.1±0.05 | 0.3±0.01* |
| Docosanol | 0.7±0.12 | 1.2±0.12* |
| 1-Hexatriacontanol | 0.7±0.15 | 0.6±0.18 |
| 1-Hexacosanol | 0.0±0.04 | 0.1±0.06 |
| 1-Octacosanol | 0.2±0.05 | 0.3±0.05 |
| 1-Triacontanol | 0.0±0.04 | 0.1±0.05 |
| 1,2,4-Butanetriol | 0.0±0.04 | 0.1±0.05 |
| Tricosanol | 0.0±0.04 | 0.1±0.05 |
| Tetracosanol | 0.1±0.10 | 0.3±0.18 |
| 1-Decanol | 0.0±0.04 | 0.0±0.00 |
| Octanediol | 0.0±0.03 | 0.0±0.00 |
| Dodecanol | 0.0±0.00 | 0.5±0.44 |
| 1-Octadecanol | 0.0±0.00 | 0.5±0.44 |
| ***Sugars*** |  |  |
| Arabinose | 0.8±0.49 | 1.9±0.70 |
| Ribose | 4.3±1.42 | 6.5±1.50 |
| Galactose | 9.8±1.41 | 12.3±1.75 |
| Glucose | 15.6±2.31 | 17.2±1.94 |
| Psicose | 2.7±0.72 | 3.1±0.87 |
| Fructose | 1.2±0.80 | 1.5±1.13 |
| Allose | 3.9±0.49 | 3.1±0.97 |
| Tagatose | 4.4±1.13 | 2.0±0.39 |
| Fucose | 2.3±1.20 | 1.5±0.48 |
| Mannose | 2.7±0.72 | 3.5±1.29 |
| Sucrose | 2.3±0.00 | 1.5±0.48 |
| Xylose | 1.6±0.78 | 0.4±0.38 |
| ***Saturated fatty acids*** |  |  |
| Butanoic acid | 1.7±0.64 | 1.7±0.81 |
| Lauric acid | 0.4±0.38 | 2.3±0.06* |
| Myristic acid | 1.1±0.47 | 1.4±0.44 |
| Palmitic Acid | 5.7±0.90 | 6.2±1.16 |
| Stearic acid | 1.1±0.13 | 2.9±0.42* |
| Behenic acid | 1.3±0.37 | 2.8±1.13 |
| Eicosanoic acid | 0.0±0.00 | 1.1±0.48 |
| Lignoceric acid | 1.5±0.47 | 1.8±0.36 |
| 4,6-Dioxoheptanoic acid | 1.3±0.91 | 0.0±0.00 |
| Azelaic acid | 0.7±0.47 | 0.3±0.34 |
| ***Unsaturated fatty acids*** |  |  |
| Oleic acid | 2.4±1.09 | 4.0±2.29 |
| Linoleic acid | 5.9±0.78 | 6.6±0.98 |
| α-linolenic acid | 2.8±0.87 | 4.6±0.63 |
| 2-Heptenoic acid, (E)- | 0.0±0.00 | 0.8±0.79 |
| Oct-3-enoic acid | 0.0±0.00 | 3.2±1.01* |
| ***Monoacylglycerols*** |  |  |
| Monopalmitin | 0.0±0.00 | 3.2±1.01 |
| Monostearin | 0.0±0.00 | 3.2±1.01 |
| Monoarachidin | 1.2±0.53 | 3.1±0.30* |
| Monomyristin | 0.4±0.35 | 1.4±0.44 |
| ***Sterols*** |  |  |
| Stigmasterol | 1.3±0.03 | 1.3±0.02 |
| β.Sitosterol | 1.0±0.50 | 1.5±0.35 |
| Stigmastanol | 1.0±0.50 | 1.5±0.35* |
| Ergost-5-ene-3,25-diol | 1.3±0.03 | 1.7±0.27 |
| Cholest-5-en-3-ol, (3.alpha.)- | 0.5±0.30 | 0.0±0.00 |
| Gorgosterol | 0.0±0.00 | 0.4±0.27 |
| ***Terpenoids*** |  |  |
| Neophytadiene | + | + |
| Citronellyl isobutyrate | + | + |
| Thymol | + | + |
| Borneol | - | + |
| ***Other compounds*** |  |  |
| Niacin | + | - |
| Uridine | + | + |
| Diacetone alcohol | + | + |
| Tocopherol | - | + |
| Ephredrine | - | + |
| Glyceryl-glycoside | + | + |

^a^ All compounds possessing hydroxy groups are identified as the correspondent TMS derivatives. Compounds were identified by comparison with the GC-MS spectral libraries NIST14.lib and WILEY229.lib; ( -) - not detected; (+)- detected; (nq) - not quantified due to the lack of an appropriate standard.

* indicates statistical significance (Mann-Whitney or t-test, p<0.05).

Supplementary Table S5 NI- non-inoculated plants; EB3 – plants inoculated with *Brevibacterium casei* EB3; RL18 – plants inoculated with *Pseudomonas oryzihabitans* RL18; EB3+RL18 –plants inoculated with *Brevibacterium casei* EB3 and *Pseudomonas oryzihabitans* RL18. Characterization of phytochemical profiles of non-inoculated and inoculated *S. europaea* grown under aquaponic microcosm conditions by ultra-performance chromatography-mass spectrometry (UHPLC-MS). Retention time (Rt; min.), wavelengths of maximum absorption in the visible region (λmax; nm), molecular ion ([M-H] ̅; m/z) and mass spectral data (MS^n^; m/z). NI - non-inoculated plants; EB3 - plants inoculated with *Brevibacterium* *casei* EB3; RL18 – plants inoculated with *Pseudomonas* *oryzihabitans* EB3+RL18 – plants inoculated with *Brevibacterium* *casei* EB3 and *Pseudomonas* *oryzihabitans*

| Rt | λ max (nm) | [M-H]^-^ | MS^2^ | NI^a^ | EB3^a^ | RL18^a^ | EB3+RL18^a^ | Assigned Identification | Reference |
| --- | --- | --- | --- | --- | --- | --- | --- | --- | --- |
| 1.36 | 208(95); 220(100) | 147 | 62 (100); 75; 85; 109; 119; 147 | 0.5±0.06 | 0.6±0.09 | 0.5±0.05 | 0.5±0.01 | Cinnamic acid | (Zhao *et al.*, 2013) |
| 1.41 | 207 | 147 | 62 (100); 75; 85; 109; 119; 147 | 0.6±0.07 | 0.6±0.09 | 0.6±0.05 | 0.6±0.02 | Cinnamic acid | (Zhao *et al.*, 2013) |
| 1.49 | 204 | 179 | 62(100); 93; 95; 96; 108; 121; 125; 137; 143; 151; 152; 161; 164; 179 | 0.6±0.07 | --- | 0.6±0.05 | 0.6±0.02 | Caffeic acid | (Wu *et al.*, 2009) |
|  | 205 | 147 | 147; 62 (100) | --- | 0.6±0.09 | --- | --- | Cinnamic acid | (Zhao *et al.*, 2013) |
| 1.62 | 254(100); 281; 319 | 163 | 163 (100); 62 | 0.6±0.07^b^ | 0.7±0.10^b^ | 0.6±0.06^b^ | 0.7±0.01^b^ | *p*-Coumaric acid | (Valente *et al.*, 2018) |
| 1.76 | 205(100); 261 | 289 | 243 (100) | 0.4±0.05 | 0.4±0.06 | 0.4±0.04 | 0.4±0.01 | Catechin | (Kang *et al.*, 2016) |
| 1.89 | 194(100); 210; 215; 245 | 295 | 85; 86; 101; 113; 119; 133; 143; 179; 187; 205; 219; 227; 237; 249; 251 (100); 255; 268; 277 | 0.5±0.07 | --- | --- | --- | Caffeoylmalic acid | (Maciejewska-Turska and Zgórka, 2022) |
|  | 192; 253 | 328 | 284 (100); 282; 240; 292; 228; 212; 178 | --- | 0.1±0.02 | 0.1±0.01 | 0.1±0.01 | Luteolin derivative | (Llorent-Martinez *et al.*, 2015) |
| 5.02 | 205; 215; 326 | 353 | 191 (100); 179 (50); 173(2); 135 (5) | 0.5±0.06 | 0.6±0.07 | 0.5±0.04 | 0.6±0.02 | 3-Caffeoylquinic acid | (Clifford *et al.*, 2003) |
| 7.34 | 309 | 175 | 62; 66; 85; 97; 100; 103; 128; 115 (100); 121; 128; 131; 133; 139; 147; 148; 157; 158; 175 | --- | nq | --- | nq | Ascorbic acid | (Szultka *et al.*, 2014) |
| 7.89 | 325(100)  241(40) | 353 | 191 (100); 179 (50); 173; 135; 335 | 0.6±0.05 | --- | 0.5±0.04 | --- | 3-Caffeoylquinic acid | (Clifford *et al.*, 2003) |
|  | 242  325(100) | 353 | 191 (100); 179 (4); 173; 161; 135; 127 | --- | 0.6±0.07 | --- | 0.7±0.08 | 5 or 1- Caffeoylquinic acid | (Clifford *et al.*, 2003) |
| 8.34 | 245(40)  298(80)  326(100) | 353 | 191 (100); 179 (41); 173 (68) | 0.5±0.06 | 0.6±0.07 | --- | --- | 3-Caffeoylquinic acid | (Clifford *et al.*, 2003) |
|  | 244  318  329 | 353 | 173 (100)  179 (61); 191 (71); 135 (5); 335 (1) | --- | --- | 0.5±0.04 | 0.6±0.02 | 4- Caffeoylquinic acid | (Clifford *et al.*, 2003) |
| 9.03 | 368; 294; 319; 335; 352 | 259 | 215 | --- | nq | --- | --- | Norbellidifodin | (Du *et al.*, 2012) |
|  | 274; 300; 311; 339 | 259(100)  387(100) | [259]: 215 (100); 191; 171  [387]: 163 (100); 119; 143; 153; 197; 207; 225 | --- | -- | nq | nq | Norbellidifodin  Medioresinol | (Du *et al.*, 2012; Molina-García *et al.*, 2018) |
| 9.48 | 253; 270 | 433 | 387 (100); 397; 396; 369; 315; 274; 255; 245 | 0.2±0.02 | 0.2±0.03 | 0.2±0.02 | 0.2±0.01 | Flavonoid derivative | (Aghakhani, Kharazian and Lori Gooini, 2018) |
| 9.64 | 311; 322; 342 | 443 | 267(100) | 0.1±0.01 | --- | --- | 0.1±0.00 | Formononetin glucoronide | (Ameixa *et al.*, 2022) |
| 9.75 | 277; 315; 332 | 225 | [225]: 225; 97(100); 136; 129; 154; 165; 181; 198; 207; 165 | nq | nq | nq | nq | Hydroxyjasmonic acid | (Valente *et al.*, 2019) |
| 10.04 | 277; 306 | 363 | 345(100); 317; 333; 305; 289;183; 257; 173; 167 | 0.4±0.19 | 0.5±0.08 | --- | 0.5±0.01 | 3-Prenyl-4-(dihydrocinnamoyloxi)-cinnamic acid | (Li and Seeram, 2018) |
| 10.10 | 277; 329; 342  369 | 389 | 119; 125; 143; 159; 164; 178; 200; 209; 227 (100); 230; 281; 299; 311; 329; 343; 345; 359 | nq | --- | --- | --- | Piceid | (Sun *et al.*, 2007) |
| 10.28 | 292; 133; 325 | 337 | 119 (100); 125; 131; 143; 149; 157; 175; 179; 218; 238; 247; 264; 271; 294; 301; 319 | 0.5±0.06 | --- | --- | --- | Ferulic acid derivative | (Spínola *et al.*, 2016) |
|  | 278; 309; 335; 368 | 321 | 101; 206; 220; 231; 241; 249; 275; 277; 291; 294; 303 (100) | --- | 0.2±0.03 | 0.2±0.02 | --- | Taxifolin | (Ameixa *et al.*, 2022) |
|  | 310 | 367 | 173 (100); 193 (8); 109 (1) | --- | --- | --- | 0.5±0.01 | 4-Feruloylquinic acid | (Clifford *et al.*, 2003) |
| 10.56 | 248; 289; 320: 330 | 443 | 267 (100); 134; 149; 161; 193; 207; 213; 231; 241; 253; 281 | 0.1±0.01 | 0.1±0.01 | 0.1±0.01 | 0.1±0.01 | Formononetin derivative | (Llorent-Martínez, Gouveia and Castilho, 2015) |
| 10.79 | 280; 292; 312; 322 | 455 | 275 (100); 179; 151; 195; 258; 289; 305; 340 | 0.1±0.08^b^ | 0.3±0.03^b^ | 0.3±0.02^b^ | 0.3±0.03^b^ | Epigallocatechin gallate | (America, no date) |
| 10.85 | 309 | 639 | 315 (100); 313; 249; 357 | 0.0±0.02 | --- | --- | --- | Isorhamnetin | Standard |
| 11.37 | 289; 314; 333; 351 | 207 | 111; 122; 125; 136; 148; 150; 163; 164; 165; 177; 180 (100); 189; 192; 207 | --- | nq | nq | nq | Sinapaldehyde | (Ghareeb *et al.*, 2018) |
| 11.43 | 264; 354 (100) | 595 | 179; 191; 235; 271; 273; 300 (78); 301 (100)  343 | 0.1±0.06 | --- | --- | --- | Quercetin derivative | (Aghakhani, Kharazian and Lori Gooini, 2018) |
| 11.65 | 282; 295; 324; 332; 343 | 355 | 337; 248 (87); 223; 311 (10); 313 (35); 310; 109; 298; 280; 217; 187; 163; 118 | 0.1±0.03 | 0.1±0.01 | 0.1±0.01 | 0.1±0.01 | Apigenin-8-C-hexoside | (Llorent-Martinez *et al.*, 2015) |
| 11.91 | 256; 354; | 463 | 301 (100); 300; 179 | 0.2±0.02 | 0.2±0.02 | 0.2±0.02 | 0.2±0.01 | Quercetin hexoside | (Valente *et al.*, 2019) |
| 12.08 | 279(10)  340(30) | 519 (100) | 337 (100); 353; 291; 181; 179; 161 | 0.5±0.06 | --- | 0.5±0.05 | 0.4±0.17 | Dicaffeoylquinic acid | (Clifford, Knight and Kuhnert, 2005) |
| 12.37 | 325(100)  293(80)  247(40) | 517 | 355 (100); 179; 173; 335; 353; 181 | 0.5±0.04 | 0.6±0.07 | 0.5±0.04 | 0.6±0.02 | Dicaffeoylquinic acid | (Clifford, Knight and Kuhnert, 2005) |
| 12.52 | 260  304 | 549 | 353 (100); 179; 193; 335 | --- | --- | 0.5±0.05 | --- | Dicaffeoylquinic acid | (Clifford, Knight and Kuhnert, 2005) |
| 12.64 | 245(50); 324(100) | 515 | 353 (100); 335 (10); 191 (4); 179 (5); 175; 173 (7) | 0.6±0.08 | 0.6±0.07 | 0.6±0.04 | 0.7±0.04 | 1,3 Dicaffeoylquinic acid | (Clifford, Knight and Kuhnert, 2005) |
| 12.86 | 327(100)  245(50) | 515 | 353 (100); 173 (1)  179 (1); 335 (1); 191 (1) | 0.7±0.07 | 0.7±0.05 | 0.6±0.03 | 1.3±0.45 | Dicaffeoylquinic acid | (Clifford, Knight and Kuhnert, 2005) |
| 13.13 | 201 (100) | 471 | 191 (100); 179 (40); 173; 161; 135 | 0.4±0.19 | --- | --- | --- | Caffeoylquinic acid derivative | (Liu *et al.*, 2018) |
|  | 251; 325 | 515 | 353 (100); 179; 191; 203; 335 | --- | 0.5±0.08 | 0.5±0.04 | 0.52+±0.01 | Dicaffeoylquinic acid | (Clifford, Knight and Kuhnert, 2005) |
| 13.25 | 250; 329 | 517.25(100)  515(60) | 355 (100); 337; 353; 173; 229; 291; 317  [515]: 353(100) | 0.3±0.17 | --- | 0.5±0.05 | --- | Dicaffeoylquinic acid | (Clifford, Knight and Kuhnert, 2005) |
| 13.51 | 246; 327 (100) | 515 | 353 (100); 299; 255; 203; 191; 179; 173; 155 | 0.7±0.09 | 0.6±0.07 | 0.6±0.03 | 0.7±0.05 | Dicaffeoylquinic acid | (Clifford, Knight and Kuhnert, 2005) |
| 13.67 | 314 (100); 249 | 515 | 353 (100); 335; 299; 255; 203; 191; 179; 173 | 0.5±0.06 | 0.6±0.08 | 0.5±0.05 | 0.5±0.02 | Dicaffeoylquinic acid | (Clifford, Knight and Kuhnert, 2005) |
| 13.83 | 328 (100); 248 | 517 | 355 (100); 337; 335; 179; 173; 181; 299; 204 | 0.5±0.05 | 0.6±0.07 | 0.5±0.04 | 0.6±0.03 | Dicaffeoylquinic acid | (Clifford, Knight and Kuhnert, 2005) |
| 14.16 | 251; 279; 329 | 515 | [515]:353 (100); 335; 255; 299; 203; 191; 179 | --- | 0.5±0.08 | 0.5±0.05 | 0.5±0.01 | Dicaffeoylquinic acid | (Clifford, Knight and Kuhnert, 2005) |
| 14.27 | 251; 277; 329; 340 | 531 | 355 (100); 337; 173; 155; 192; 213; 323; 349; 369 | 0.5±0.06 | --- | --- | --- | Dicaffeoylquinic acid | (Clifford, Knight and Kuhnert, 2005) |
| 14.69 | 249; 278; 332; 357 | 515 | 353 (100); 335; 299; 265; 255; 203; 191; 173 | --- | --- | 0.5±0.05 | 0.5±0.01 | Dicaffeoylquinic acid | (Clifford, Knight and Kuhnert, 2005) |
| 14.81 | 251; 280; 313; 348 | 429 (100)  267 | 285; 267; 249(100); 241; 205; 179; 161 | --- | 0.1±0.01 | 0.1±0.01 | 0.1±0.00 | Ononin (Formononetin 7-O-glucoside) | (America, no date) |
| 15.02 | 251; 279; 313; 320 | 429 | 249 (100); 205; 241; 161; 179; 187; 195; 267 | --- | 0.1±0.01 | 0.1±0.01 | 0.1±0.00 | Ononin (Formononetin 7-O-glucoside) | (America, no date) |
| 15.41 | 251; 279; 320 | 390 (100)  267 | **[390]:** 278 (100); 283 (42); 259 (61); 173 (46)  **[267]:** 97 (100); 223 (99); 240; 249; 205 | --- | 0.1±0.01 | 0.1±0.01 | 0.1±0.00 | Formononetin derivative | (Kang, Hick and Price, 2007) |
| 15.97 | 327 (100); 248; 368 | 619 | 443 (100); 267 (4) | 0.1±0.01 | 0.1±0.01 | 0.1±0.01 | 0.1±0.01 | Formononetin derivative | (Llorent-Martínez, Gouveia and Castilho, 2015) |
| 16.59 | 278; 337; 384 | 327 | 291 (100); 297 (10); 201 (59); 265 (20); 252 (15); 229 (11);  209(7); 171 (64) | --- | nq | nq | nq | Oxo-dihydroxy- octadecenoic acid | (Llorent-Martínez, Gouveia and Castilho, 2015) |
| 16.88 | 311 | 404 | [404]: 324 (100); 158; 164; 186; 192; 273; 290; 306; 316 | --- | nq | nq | nq | Unknown |  |
| 16.96 | 298 | 794 | 632.46 (100)  674.55 | nq | --- | --- | --- | unknown |  |
| 17.10 | 289; 337; 353 | 619 | 443 (100) | 0.1±0.00 | --- | --- | --- | Formononetin derivative | (Llorent-Martínez, Gouveia and Castilho, 2015) |
|  | 251; 314; 339 | 547 | 193 (100); 249(78.5); 161 (8); 281(8); 245 (7); 241 (5.5); 253 (4) | --- | 0.5±0.08 | --- | --- | Ferulic acid derivative | (Spínola *et al.*, 2016) |
|  | 285; 318; 328; 339; 354 | 547 | 249 (100); 193 (90); 353(40); 175; 191; 337; 161 | --- | --- | 0.5±0.05 | 0.5±0.02 | Caffeoyferuloyoylquinic acid derivative | (Clifford *et al.*, 2003) |
| 17.33 | 289 | 209 | 97 (90); 209 (100); 112; 167; 181; 191; 194 | 0.5±0.06 | 0.6±0.09 | 0.5±0.05 | 0.5±0.01 | Hydroxyferulic acid | (Huang *et al.*, 2015) |
| 18.45 | 276; 314; 335; 356 | 475 | 241; 275; 293 (100); 295; 313; 351; 390; 405 | --- | --- | --- | 0.1±0.00 | 5-Hydroxyl-6,8-dimethoxy-7-hexoside flavone | (Gouveia and Castilho, 2010) |

^a^ mg of compound/g plant dry weight±SE (mg g^-1^DW), using three replicates for the error calculation; nq = non quantified due to the lack of the appropriate standard or because it is an unknown compound. ^b^ standard solution documented at 280 nm

Supplementary Table S6. Characterization of phytochemical profiles of non-inoculated and inoculated *S. europaea* grown under aquaponic pilot-scale conditions by ultra-performance chromatography-mass spectrometry (UHPLC-MS). Retention time (Rt; min.), wavelengths of maximum absorption in the visible region (λmax; nm), molecular ion ([M-H] ̅; m/z) and mass spectral data (MS^n^; m/z). NI - non-inoculated plants; EB3+RL18 – plants inoculated with *Brevibacterium* *casei* EB3 and *Pseudomonas* *oryzihabitans.*

| Rt | λ max (nm) | [M-H]^-^ | MS^2^ | NI^a^ | EB3+RL18^a^ | Assigned Identification | REF |
| --- | --- | --- | --- | --- | --- | --- | --- |
| 1.36 | 241; 327 (100) | 147 | 62 (100); 75; 119; 17 (90) | 0.5±0.06 | --- | Cinnamic acid | (Zhao *et al.*, 2013) |
|  | 205 | 215 | 95 (100); 97 (99); 100;118; 147; 153; 157; 161; 173; 177; 179; 185; 197; 215 | --- | 0.5±0.04 | Caffeic acid derivative | (Gouveia and Castilho, 2010) |
| 1.41 | 241; 327 (100) | 147 | 62 (100); 75; 119; 17 (90) | 0.5±0.07 | --- | Cinnamic acid | (Zhao *et al.*, 2013) |
|  | 205 | 215 | 95 (100); 97 (99); 100;118; 147; 153; 157; 161; 173; 177; 179; 185; 197; 215 | --- | 0.5±0.05 | Caffeic acid derivative | (Gouveia and Castilho, 2010) |
| 1.50 | 193(100) | 147 | 62 (100); 75; 101; 119; 129; 147 | 0.5±0.07 | --- | Cinnamic acid | (Zhao *et al.*, 2013) |
|  | 190 (100)  256 | 215 | 95 (90); 97 (100); 113; 127; 129; 131; 141; 143; 147; 157; 171; 173; 179; 187; 197; 215 | --- | 0.5±0.05 | Caffeic acid derivative | (Gouveia and Castilho, 2010) |
| 1.63 | 254(100); 281(75); 319(45); 375(35) | 163 | [163]: 95 (100); 163 (90); 62; 75; 89; 93; 105; 119; 135; 145; 164 | 0.6±0.07 | 0.6±0.06 | *p*-Coumaric acid | (Valente *et al.*, 2018) |
| 1.76 | 203(100); 263(30) | 289 | [289]: 243(100); 253; 229; 213; 215; 221; 194; 172; 154 | 0.4±0.05^b^ | 0.4±0.04^b^ | Catechin | Standard |
| 5.06 | 206 (100) | 353 | 191 (100); 179 (46); 173 (2); 135 (10) | 0.5±0.06 | 0.5±0.04 | 3-Caffeoylquinic acid | (Clifford *et al.*, 2003) |
| 7.92 | 241(40)  327(100) | 353 | 191 (100); 179 (23); 173 (36); 135 (3)  335 | 0.5±0.05 | --- | 3-Caffeoylquinic acid | (Clifford *et al.*, 2003) |
|  | 242; 273; 310; 320; 331 | 353 | 191 (100); 179 (8); 173 (5); 135 (1.3)  155; 161; 335 | --- | 1.0±0.04 | 5 or 1- Caffeoylquinic acid | (Clifford *et al.*, 2003) |
| 8.38 | 271; 300; 321; 331; 339 | 353 | 191 (100); 179 (34); 173 (50); 135 (2)  335 (2) | 0.5±0.06 | 0.5±0.04 | 3-Caffeoylquinic acid | (Clifford *et al.*, 2003) |
| 8.68 | 267; 273; 308; 327; 344; 355 | 305 | 97; 178; 205; 215; 221; 225 (100); 233; 242; 248; 259; 262; 285; 287; 289 | --- | 0.2±0.02^b^ | Gallocatechin | (Ameixa *et al.*, 2022) |
| 9.32 | 244; 301; 344; 355 | 305 | 97; 225; 165; 188; 205; 221; 241; 265 | --- | 0.2±0.02^b^ | Gallocatechin | (Ameixa *et al.*, 2022) |
| 9.51 | 245; 262; 273 | 433 | 161; 170; 293; 301 (30); 318; 345; 353; 370; 387 (100); 397; 401; 415; 433 | --- | 0.2±0.02 | Quercetin derivative | (Aghakhani, Kharazian and Lori Gooini, 2018) |
| 9.68 | 273; 301; 308; 336; 348 | 433 | 161; 170; 293; 301 (62); 318; 345; 353; 370; 387 (100); 397; 401; 415; 433 | --- | 0.2±0.01 | Quercetin derivative | (Aghakhani, Kharazian and Lori Gooini, 2018) |
| 10.07 | 282; 292; 299; 307 | 137 | 66; 79; 82; 91; 95; 109; 110; 111; 119; 122; 137 (100) | --- | 0.5±0.04 | Hydroxybenzoic acid |  |
| 10.60 | 293; 310; 332; 340 | 443 | 135; 171; 193; 200; 217; 240; 249; 253; 267 (100); 301; 312; 329; 357; 381; 411; 425; 426 | 0.1±0.01 | 0.1±0.01 | Formononetin glucuronide | (Leng *et al.*, 2022) |
| 10.81 | 282; 292; 301; 311; 319 | 163 |  | --- | 0.5±0.04 | *p*-Coumaric acid | (Valente *et al.*, 2018) |
| 10.97 | 263; 311; 339; 355 | 433 | 180; 201. 207; 221; 225; 267 (100) | --- | 0.1±0.01 | Formononetin derivative | (Llorent-Martínez, Gouveia and Castilho, 2015) |
| 11.45 | 290; 297; 307; 355 | 163 | 163 | --- | 0.5±0.04 | p-coumaric acid | (Valente *et al.*, 2018) |
| 12.13 | 276; 314; 336; 353 | 509 | 241 (100) | --- | 0.1±0.01 | Luteolin derivative | (Spínola *et al.*, 2016) |
| 12.21 | 292; 311; 331; 340 | 449 | 269 (100) | --- | 0.1±0.06 | Kaempferol derivative | (Kang *et al.*, 2016) |
| 12.42 | 246; 325(100) | 517(100) | 355 (100); 337; 335; 353; 301; 203; 179; 151 | 0.5±0,06 | 0.5±0.04 | Dicaffeoylquinic acid | (Clifford, Knight and Kuhnert, 2005) |
| 12.68 | 245(50); 325(100) | 515 | [515]: 353 (100); 335; 255; 241; 203; 191; 179; 161 | 0.5±0.07 | 0.6±0.03 | Dicaffeoylquinic acid | (Clifford, Knight and Kuhnert, 2005) |
| 12.91 | 244(50); 327(100) | 515 | [515]: 353 (100); 335; 203; 173 | 0.6±0.11 | 0.6±0.02 | Dicaffeoylquinic acid | (Clifford, Knight and Kuhnert, 2005) |
| 13.20 | 249; 271; 307; 325; 336 | 429 | 267 (100); 249; 243; 233 | --- | 0.1±0.01 | Formononetin derivative | (Kang *et al.*, 2016) |
| 13.30 | 250; 281; 321; 335; 350 | 517(100) | 355; 353; 301; 267; 255; 203; 191; 173; 157 | 0.5±0.06 | --- | Dicaffeoylquinic acid | (Clifford, Knight and Kuhnert, 2005) |
| 13.55 | 246(50); 327(100) | 515(100) | 353 (100); 299; 255; 203; 191; 179 | 0.6±0.08 | 0.6±0.04 | Dicaffeoylquinic acid | (Clifford, Knight and Kuhnert, 2005) |
| 13.70 | 250; 314 | 515 | 173 (0.28); 179 (4); 191 (6); 203 (9); 299 (10); 335 (8); 353 (100) | 0.5±0.06 | 0.5±0.07 | 1,3 Dicaffeoylquinic acid | (Clifford, Knight and Kuhnert, 2005) |
| 13.87 | 331 | 517 | [517]: 355 (100); 337; 299; 203; 181; 179; 173; 155 | 0.5±0.06 | 0.5±0.04 | Dicaffeoylquinic acid | (Clifford, Knight and Kuhnert, 2005) |
| 14.19 | 310(45); 18(50); 328(45) | 163 |  | --- | 0.5±0.04 | *p*-Coumaric acid | (Valente *et al.*, 2018) |
| 14.31 | 310(45); 18(50); 328(45) | 163 |  | 0.5±0.06 | --- | *p*-Coumaric acid | (Valente *et al.*, 2018) |
|  | 249; 281; 319; 330 | 531 | 175; 178; 191; 193 (100); 207; 221; 235; 243; 257 | --- | 0.5±0.04 | Feruloylquinic acid derivative | (Clifford *et al.*, 2003) |
| 14.55 | 251; 290; 316; 339 | 283 | 133; 143; 153; 209; 211; 239; 253 (100); 254; 268 | --- | 0.1±0.01 | Acacetin | (Falcão *et al.*, 2013) |
| 14.87 | 251; 288; 309; 317; 328 | 430 | 161; 205; 206; 241; 250 (100); 265 | --- | 0.5±0.04 | 1,3-*O*-Feruloyl-caffeoylglycerol | (Kang *et al.*, 2016) |
| 15.06 | 250; 283; 301; 318; 330; 339 | 429 | 135; 143; 161; 165; 172; 179; 187; 205; 229; 241; 249 (100); 265 | --- | 0.5±0.04 | 1,3-*O*-Feruloyl-caffeoylglycerol | (Kang *et al.*, 2016) |
| 15.32 | 291; 318; 328 | 676 (50)  691(50) | **[676]:** 448; 458; 497; 514; 632; 644; 649  **[691]:** 337 (100); 352; 515; 597; 673 | nq | --- | unknown |  |
|  | 251; 288 | 691 | 279; 325; 340; 471; 511; 528; 545; 575; 646; 673 (100) | --- | nq | unknown |  |
| 15.48 | 282; 317; 328 | 390 | [390]: 310 (100) | nq | --- | unknown |  |
|  | 260; 278; 291; 318; 327 | 267 | 97; 223 (100) | --- | 0.1±0.01 | Formononetin | (Llorent-Martínez, Gouveia and Castilho, 2015) |
| 15.92 | 251; 287; 316; 324 | 619 | 443 (100); 267 | ---- | 0.1±0.01 | Formononetin derivative | (Llorent-Martínez, Gouveia and Castilho, 2015) |
| 16.00 | 250; 301; 325; 331; 340 | 619 | [619]: 239; 267; 283; 307; 353; 381; 383; 443 (100) | 0.1±0.01 | 0.1±0.01 | Formononetin derivative | (Llorent-Martínez, Gouveia and Castilho, 2015) |
| 16.52 | 283; 294; 312; 324 | 673 | 223; 250; 299; 337; 350; 417; 433; 443; 471; 482; 511; 530; 585; 597; 611; 630 (100); 641; 655 (99) | --- | nq | unknown |  |
| 16.64 | 279; 289; 307; 329 | 605 | 181; 423; 429; 443; 454; 480; 521; 557; 561 (100); 569; 587 | ---- | nq | unknown |  |
| 17.19 | 291; 326; 334 | 329 | 171; 201 (100); 275; 293; 311; 327 | ---- | nq | Trihydroxy-octadecenoic acid | (Llorent-Martínez, Gouveia and Castilho, 2015) |
| 17.38 | 290 | 479 | 239; 241; 257 (100) | 0.1±0.01 | --- | Flavone derivative | Standard |
|  | 291 | 163 | 62; 82; 95; 98; 120; 121 (100); 136; 146; 148; 163 | --- | 0.5±0.05 | *p*-Coumaric | (Valente *et al.*, 2018) |
| 17.90 | 274; 294; 317; 334; 352 | 327 | 171; 183; 193; 209; 211; 229 (100); 239; 247; 273; 289; 291; 309 | --- | nq | Oxo-dihydroxy- octadecenoic acid | (Llorent-Martínez, Gouveia and Castilho, 2015) |
| 18.21 | 287; 317; 351 | 327 | 171; 183; 185; 201; 209; 211; 221; 229; 239; 247; 257; 273; 289; 291 (100); 307; 309; 327 | --- | nq | Oxo-dihydroxy- octadecenoic acid | (Mahomoodally *et al.*, 2020) |

^a^ mg of compound/g plant dry weight±SE (mg g^-1^ DW), using three replicates for the error calculation; nq = non quantified due to the lack of the appropriate standard or because it is an unknown compound. ^b^ standard solution documented at 280 n

References

Aghakhani, F., Kharazian, N. and Lori Gooini, Z. (2018) ‘Flavonoid Constituents of Phlomis (Lamiaceae) Species Using Liquid Chromatography Mass Spectrometry’, *Phytochemical Analysis*, 29(2), pp. 180–195. Available at: https://doi.org/10.1002/pca.2733.

Ameixa, O.M.C.C. *et al.* (2022) ‘Gall midge Baldratia salicorniae Kieffer (Diptera: Cecidomyiidae) infestation on Salicornia europaea L. induces the production of specialized metabolites with biotechnological potential’, *Phytochemistry*, 200(March). Available at: https://doi.org/10.1016/j.phytochem.2022.113207.

America, M. of N. (no date) *MassBank: The database of high-resolution mass spectra*.

Clifford, M.N. *et al.* (2003) ‘Hierarchical scheme for LC-MSn identification of chlorogenic acids’, *Journal of Agricultural and Food Chemistry*, 51(10), pp. 2900–2911. Available at: https://doi.org/10.1021/jf026187q.

Clifford, M.N., Knight, S. and Kuhnert, N. (2005) ‘Discriminating between the six isomers of dicaffeoylquinic acid by LC-MSn’, *Journal of Agricultural and Food Chemistry*, 53(10), pp. 3821–3832. Available at: https://doi.org/10.1021/jf050046h.

Du, X.G. *et al.* (2012) ‘Identification of xanthones from Swertia punicea using high-performance liquid chromatography coupled with electrospray ionization tandem mass spectrometry’, *Rapid Communications in Mass Spectrometry*, 26(24), pp. 2913–2923. Available at: https://doi.org/10.1002/rcm.6419.

Falcão, S.I. *et al.* (2013) ‘Phenolic quantification and botanical origin of Portuguese propolis’, *Industrial Crops and Products*, 49, pp. 805–812. Available at: https://doi.org/10.1016/j.indcrop.2013.07.021.

Ghareeb, M. *et al.* (2018) ‘HPLC-DAD-ESI-MS/MS characterization of bioactive secondary metabolites from Strelitzia nicolai leaf extracts and their antioxidant and anticancer activities in vitro’, *Pharmacognosy Research*, 10(4), pp. 368–378. Available at: https://doi.org/10.4103/pr.pr_89_18.

Gouveia, S.C. and Castilho, P.C. (2010) ‘Characterization of phenolic compounds in Helichrysum melaleucum by high-performance liquid chromatography with on-line ultraviolet and mass spectrometry detection’, *Rapid Communications in Mass Spectrometry*, 24(13), pp. 1851–1868. Available at: https://doi.org/https://doi.org/10.1002/rcm.4585.

Huang, R.T. *et al.* (2015) ‘Determination of phenolic acids and flavonoids in Rhinacanthus nasutus (L.) kurz by high-performance-liquid-chromatography with photodiode-array detection and tandem mass spectrometry’, *Journal of Functional Foods*, 12, pp. 498–508. Available at: https://doi.org/10.1016/j.jff.2014.12.002.

Kang, J. *et al.* (2016) ‘Identification and characterization of phenolic compounds in hydromethanolic extracts of sorghum wholegrains by LC-ESI-MSn’, *Food Chemistry*, 211, pp. 215–226. Available at: https://doi.org/10.1016/j.foodchem.2016.05.052.

Kang, J., Hick, L.A. and Price, W.E. (2007) ‘A fragmentation study of isoflavones in negative electrospray ionization by MSn ion trap mass spectrometry and triple quadrupole mass spectrometry’, *Rapid Communications in Mass Spectrometry*, 21(6), pp. 857–868. Available at: https://doi.org/10.1002/rcm.2897.

Leng, Z. *et al.* (2022) ‘Identification of Phenolic Compounds in Australian-Grown Bell Peppers by Liquid Chromatography Coupled with Electrospray Ionization-Quadrupole-Time-of-Flight-Mass Spectrometry and Estimation of Their Antioxidant Potential’, *ACS Omega*, 7(5), pp. 4563–4576. Available at: https://doi.org/10.1021/acsomega.1c06532.

Li, C. and Seeram, N.P. (2018) ‘Ultra-fast liquid chromatography coupled with electrospray ionization time-of-flight mass spectrometry for the rapid phenolic profiling of red maple (Acer rubrum) leaves’, *Journal of Separation Science*, 41(11), pp. 2331–2346. Available at: https://doi.org/10.1002/jssc.201800037.

Liu, L. *et al.* (2018) ‘Rapid characterization of chlorogenic acids in Duhaldea nervosa based on ultra-high-performance liquid chromatography–linear trap quadropole-Orbitrap-mass spectrometry and mass spectral trees similarity filter technique’, *Journal of Separation Science*, 41(8), pp. 1764–1774. Available at: https://doi.org/10.1002/jssc.201701047.

Llorent-Martinez, E.J. *et al.* (2015) ‘HPLC-ESI-MSn characterization of phenolic compounds, terpenoid saponins, and other minor compounds in Bituminaria bituminosa’, *Industrial Crops and Products*, 69, pp. 80–90. Available at: https://doi.org/10.1016/j.indcrop.2015.02.014.

Llorent-Martínez, E.J., Gouveia, S. and Castilho, P.C. (2015) ‘Analysis of phenolic compounds in leaves from endemic trees from Madeira Island. A contribution to the chemotaxonomy of Laurisilva forest species’, *Industrial Crops and Products*, 64, pp. 135–151. Available at: https://doi.org/10.1016/j.indcrop.2014.10.068.

Maciejewska-Turska, M. and Zgórka, G. (2022) ‘In-depth phytochemical and biological studies on potential AChE inhibitors in red and zigzag clover dry extracts using reversed–phase liquid chromatography (RP-LC) coupled with photodiode array (PDA) and electron spray ionization-quadrupole/time of flight’, *Food Chemistry*, 375, p. 131846. Available at: https://doi.org/https://doi.org/10.1016/j.foodchem.2021.131846.

Mahomoodally, M.F. *et al.* (2020) ‘Phytochemical analysis, network pharmacology and in silico investigations on anacamptis pyramidalis tuber extracts’, *Molecules*, 25(10). Available at: https://doi.org/10.3390/molecules25102422.

Molina-García, L. *et al.* (2018) ‘Determination of the Phenolic Profile and Antioxidant Activity of Leaves and Fruits of Spanish Quercus coccifera’, *Journal of Chemistry*, 2018. Available at: https://doi.org/10.1155/2018/2573270.

Spínola, V. *et al.* (2016) ‘Ulex europaeus: From noxious weed to source of valuable isoflavones and flavanones’, *Industrial Crops and Products*, 90, pp. 9–27. Available at: https://doi.org/10.1016/j.indcrop.2016.06.007.

Sun, J. *et al.* (2007) ‘Screening Non-colored Phenolics in Red Wines using Liquid Chromatography/Ultraviolet and Mass Spectrometry/Mass Spectrometry Libraries’, *Molecules*, 12(3), pp. 679–693. Available at: https://doi.org/10.3390/12030679.

Szultka, M. *et al.* (2014) ‘Determination of ascorbic acid and its degradation products by high-performance liquid chromatography-triple quadrupole mass spectrometry’, *Electrophoresis*, 35(4), pp. 585–592. Available at: https://doi.org/10.1002/elps.201300439.

Valente, I.M. *et al.* (2018) ‘Profiling of phenolic compounds and antioxidant properties of European varieties and cultivars of Vicia faba L. pods’, *Phytochemistry*, 152, pp. 223–229. Available at: https://doi.org/10.1016/j.phytochem.2018.05.011.

Valente, I.M. *et al.* (2019) ‘Unravelling the phytonutrients and antioxidant properties of European Vicia faba L. seeds’, *Food Research International*, 116, pp. 888–896. Available at: https://doi.org/https://doi.org/10.1016/j.foodres.2018.09.025.

Wu, Z.J. *et al.* (2009) ‘Analysis of caffeic acid derivatives from Osmanthus yunnanensis using electrospray ionization quadrupole time-of-flight mass spectrometry’, *European Journal of Mass Spectrometry*, 15(3), pp. 415–429. Available at: https://doi.org/10.1255/ejms.992.

Zhao, H.Y. *et al.* (2013) ‘Chemical profiling of the chinese herb formula Xiao-Cheng-Qi decoction using liquid chromatography coupled with electrospray ionization mass spectrometry’, *Journal of Chromatographic Science*, 51(3), pp. 273–285. Available at: https://doi.org/10.1093/chromsci/bms138.
